# Efficacy of lower limb strengthening exercises based on different muscle contraction characteristics for knee osteoarthritis: a systematic review and network meta-analysis

**DOI:** 10.3389/fmed.2024.1442683

**Published:** 2024-09-25

**Authors:** Xiaoqing Ding, Yi Yang, Ying Xing, Qingsong Jia, Qingguo Liu, Jie Zhang

**Affiliations:** ^1^Heilongjiang University of Chinese Medicine, Harbin, China; ^2^School of Acupuncture-Moxibustion and Tuina, Beijing University of Chinese Medicine, Beijing, China; ^3^The Second Affiliated Hospital of Heilongjiang University of Chinese Medicine, Harbin, China

**Keywords:** strengthening exercise, isotonic exercise, isometric exercise, isokinetic exercise, knee osteoarthritis

## Abstract

**Purpose:**

While strengthening exercises are recommended for knee osteoarthritis (KOA) treatment, the optimal type of muscle contraction remains unclear, with current research showing conflicting results. This network meta-analysis (NMA) aims to evaluate the efficacy of lower limb strengthening exercises based on different muscle contraction characteristics for KOA patients and provide clinical references.

**Methods:**

We conducted the NMA following the PRISMA-NMA. A comprehensive search of five databases (PubMed, Web of Science, CENTRAL, Embase, and SPORTDiscus) up to August 2024 identified randomized controlled trials (RCTs) investigating lower limb strengthening exercises in KOA patients. Control groups included receiving usual care, only providing health education, or no intervention at all. Outcomes analyzed included pain, physical function, quality of life, and muscle strength.

**Results:**

Forty-one studies (2,251 participants) were included. Twenty-eight studies used rigorous randomization; eighteen reported allocation concealment. All had high performance bias risk due to exercise interventions. Regarding efficacy, isokinetic exercise ranked highest in pain relief (SMD = 0.70, 95% CI: 0.50–0.91, SUCRA = 82.6%), function improvement (SMD = 0.75, 95% CI: 0.57–0.92, SUCRA = 96.1%), and enhancement in muscle strength (SMD = 0.56, 95% CI: 0.34–0.78, SUCRA = 90.1%). Isometric exercise ranked highest in improving quality of life (SMD = 0.80, 95% CI: 0.28–1.31, SUCRA = 90.5%). Mixed strengthening exercise ranked lowest across all outcomes. High-frequency interventions (≥5 times/week) showed superior pain relief compared with low-frequency (≤3 times/week) for isotonic, isometric, and isokinetic exercise.

**Conclusion:**

This NMA suggests isokinetic exercise may be most effective for pain, function, and muscle strength in KOA patients, while isometric exercise benefits quality of life most. Mixed strengthening exercise ranked lowest across all outcomes. High-frequency interventions appear more effective than low-frequency ones. These findings support personalized KOA treatment, considering efficacy, accessibility, and patient-specific factors. Study biases, heterogeneity, and other limitations may affect result reliability. Future research should focus on high-quality studies with standardized protocols and analyze dose–response relationships to refine KOA treatment strategies.

**Systematic review registration:**

https://www.crd.york.ac.uk/prospero/display_record.php?ID=CRD42024582525, identifier: CRD42024582525.

## 1 Introduction

Knee osteoarthritis (KOA) is a prevalent degenerative joint disease, manifesting as pain, stiffness, and functional limitations, frequently resulting in physical disability. Among these symptoms, chronic pain, the primary manifestation, substantially impacts patients’ daily activities, mental well-being, and overall quality of life (1). These symptoms arise from a complex interplay of factors, including cartilage degradation, bone remodeling, synovial inflammation, ligament dysfunction, muscle atrophy, and periarticular fat alterations. This is further exacerbated by age, obesity, and psychological factors (2, 3). KOA represents a significant global public health burden. The 2021 Global Burden of Disease Study reported an age-standardized prevalence of 4,307.4 cases per 100,000 in 2020, with projections of 642 million people affected by 2050 (4). In China, KOA cases surged 153.98% from 1990 to 2019, reaching 108.12 million, with further growth expected by 2044 (5). In the United States, KOA imposes substantial economic burdens, with direct medical costs ranging from $1,227 to $19,530 per patient (6).

KOA treatment encompasses non-pharmacological approaches (exercise, education, weight loss), pharmacological interventions (NSAIDs, intra-articular injections), and surgical procedures (primarily total knee replacement for advanced cases) (2). Long-term medication use, although providing relief of symptoms, can also lead to gastrointestinal issues, cardiovascular risks, and liver toxicity (7). A meta-analysis showed adverse events in 29.8% of NSAID users and 89.5% of opioid users (8). While recommended for end-stage KOA, surgery poses substantial risks and costs, limiting its suitability for all patients (9). Exercise therapy is widely acknowledged as a safe, effective, and cost-efficient non-pharmacological intervention. Several systematic reviews showed exercise therapy can significantly reduce pain, improve function, and enhance the quality of life for KOA patients (10–13).

The 2019 American College of Rheumatology/Arthritis Foundation Guideline recommends a range of exercises, including aerobic, strengthening, neuromuscular, aquatic, and balance exercises (14). Strengthening exercises have recently garnered attention for their potential benefits in KOA treatment. Research shows that the efficacy in pain reduction and functional improvement with strengthening exercises is comparable to that of aerobic, balance, and neuromuscular training (15–19). Compared to aquatic exercises, strengthening exercises demonstrate superior performance in alleviating joint stiffness (20). Strengthening exercises are recommended for KOA treatment as they not only alleviate symptoms but also address muscle weakness, a common issue in KOA patients, which may help prevent functional limitations in early-stage KOA (21, 22). Other interventions, such as neuromuscular training and balance exercises, may not directly target muscle weakness.

While the benefits of strengthening exercises in KOA treatment are well established, an important question remains: what is the optimal type of muscle contraction during these exercises? Typically, strengthening exercises are categorized into isotonic, isometric, and isokinetic types based on the characteristics of muscle contraction (23, 24). These contraction methods may affect joint loading, pain modulation, and functional improvement in KOA patients in different ways, potentially yielding varied outcomes. Current research shows mixed findings. Salli et al. found isokinetic exercise to be more effective than isometric exercise in relieving pain and improving function, while Çakır et al. reported no significant difference between the two (25, 26). Given these conflicting findings and lack of comprehensive comparisons across different contraction types, this study employs a network meta-analysis (NMA) to evaluate the efficacy of lower limb strengthening exercises with different muscle contraction types for KOA patients, aiming to provide a reference for clinical settings.

## 2 Methods

This study adheres to the Preferred Reporting Items for Systematic Reviews and Meta-Analyses for Network Meta-Analyses (PRISMA-NMA) (27), and is registered with PROSPERO (CRD42024582525).

### 2.1 Search strategy

To comprehensively evaluate the efficacy of various lower limb strengthening exercise modalities for KOA treatment, we will conduct a systematic search of PubMed, Web of Science, Cochrane Central Register of Controlled Trials (CENTRAL), Embase, and SPORTDiscus databases for relevant randomized controlled trials (RCTs) up to August 2024. We will also search PubMed and Embase for systematic reviews and meta-analyses published between 2019 and 2024. Relevant studies from these reviews will be extracted and integrated into our initial search results. EndNote X9 will be employed for literature management, screening, and duplicate removal. Three researchers (X.D., Y.Y., and Y.X.) will independently perform preliminary screening based on titles and abstracts, adhering to the inclusion and exclusion criteria. Subsequent full-text reviews will determine final inclusion. Any discrepancies will be resolved through consensus discussions among the researchers. Detailed search strategies and corresponding formulas are provided in Supplementary Appendix 1.

### 2.2 Study selection and eligibility

#### 2.2.1 Types of studies

Inclusion criteria will be limited to RCTs published in peer-reviewed journals. We will exclude cross-sectional studies, animal experiments, systematic reviews, meta-analyses, and clinical guidelines. For studies with inaccessible full texts, we will contact the corresponding authors via email for assistance. To mitigate potential participant overlap in articles from the same research team, we will meticulously examine study methods and participant characteristics. When necessary, we will seek author clarification. In cases of confirmed overlap, we will include only the study with the largest sample size or most comprehensive relevant data. For studies with multiple published versions, we will select the most recent iteration.

#### 2.2.2 Types of participants

The study population will comprise adults (aged ≥18 years) diagnosed with KOA, irrespective of gender or race. The exclusion criteria include patients without a definitive KOA diagnosis, those who have undergone KOA-related surgery, and individuals with other serious conditions that may affect lower limb function (e.g., severe osteoporosis or rheumatoid arthritis).

#### 2.2.3 Types of interventions

To assess the distinct impacts of different strengthening exercise modalities, this study will incorporate interventions focused solely on lower limb strengthening exercises. Additionally, it will include interventions where the experimental group undergoes strengthening exercises in conjunction with usual care, while the control group receives usual care alone. The study design will impose no limitations on instructional methodologies, exercise frequencies, or intervention durations. Lower limb strengthening exercises will be classified according to muscle contraction characteristics into four categories: isotonic, isometric, isokinetic, and mixed strengthening exercise (incorporating two or more of the aforementioned types). Comprehensive definitions for each exercise category are delineated in Supplementary Appendix 2.

#### 2.2.4 Types of comparisons

The control group will comprise participants from the following categories: (a) Those receiving usual care from healthcare professionals, including pharmacological treatments (e.g., NSAIDs) and non-exercise local physical therapies (e.g., hot packs, interferential current therapy); (b) Those not receiving specific interventions (e.g., waiting list participants or those maintaining normal daily activities); and (c) Those receiving only health education (e.g., information on disease management, lifestyle adjustments, and self-care strategies). The control group will exclude any structured exercise intervention programs.

#### 2.2.5 Types of outcomes

Pain will serve as the primary outcome measure, with physical function, quality of life, and muscle strength serving as secondary outcomes. The selection of pain, physical function, and quality of life is based on recommended outcomes for OA trials (28, 29). Muscle strength is included as an outcome measure due to its direct relevance to lower limb strengthening exercises. For studies using multiple scales to assess pain, function, or quality of life, the most comprehensively reported scale will be selected based on the ranking order by French et al. (30). Muscle strength parameters will be prioritized as follows: knee extensors, knee flexors, followed by other muscle groups. For trials reporting multiple intensities, results from the highest intensity will be selected. The primary time reference point will be the end of each study’s intervention period.

### 2.3 Data extraction

A custom data extraction form will be designed to capture key information from included studies. The form will capture study identifiers including first authors’ names, publication year, and registration number. It will also record participant count, demographics (age, gender), intervention details (including frequency, duration, and professional supervision), and key outcome metrics. Two independent researchers (X.D. and Q.J.) will screen and extract the data. Disagreements will be resolved through discussion or consensus with other team members. Extracted outcome data will include pre- and post-intervention means and standard deviations. Data reported as median, range, or other formats will be converted to mean and standard deviation using established statistical methods, such as those by Hozo et al. (31).

### 2.4 Quality assessment

Three independent researchers (Y.Y., Y.X., and J.Z.) will assess study bias risk using the Cochrane Collaboration’s risk of bias tool (32). The tool evaluates several bias categories: including selection (random sequence generation, allocation concealment), performance (participant and personnel blinding), detection (outcome assessment blinding), attrition (incomplete outcome data), reporting (selective reporting), and other sources. Each bias category will be rated as low, high, or unclear risk. Due to the nature of exercise training interventions, patient blinding is challenging. Therefore, all studies will be deemed at high risk of performance bias. Disagreements in quality assessment will be resolved through researcher discussion.

### 2.5 Statistical analysis

#### 2.5.1 Features of NMA

NMA extends pairwise meta-analysis by enabling simultaneous comparison of multiple interventions, incorporating both direct and indirect evidence for comprehensive synthesis. The network evidence graph, a key NMA component, depicts interventions as nodes and direct comparisons as edges, with their sizes reflecting evidence volume. NMA reliability hinges on consistency between direct and indirect evidence, assessed at both global and local levels. Global consistency evaluates network-wide agreement, while local consistency examines specific comparison loops. NMA notably ranks treatments by relative effectiveness, often using the Surface Under the Cumulative Ranking curve (SUCRA), which quantifies each intervention’s cumulative ranking probability (33). Comparison-adjusted funnel plots are used to detect potential publication bias by visualizing small-study effects and network asymmetries. These methods collectively establish NMA as a crucial tool for informing evidence-based clinical decision-making.

#### 2.5.2 Implementation of NMA

The NMA will adhere to PRISMA NMA guidelines (34). Initially, a network evidence map will be constructed to visualize relationships between various lower limb strengthening exercise modalities. Inconsistent NMA models will be fitted, with global inconsistency assessed via the Wald test and local inconsistency via the node-splitting method. The consistency model will be prioritized if consistency is good; otherwise, the inconsistency model will be used, and potential sources of inconsistency explored. For reverse-scaled studies (where lower values indicate better outcomes), group means will be multiplied by −1, as recommended by the Cochrane Handbook (32). Standardized mean difference (SMD) will be used as the effect size for continuous outcomes, enabling comparison and synthesis across different scales. SUCRA will be used to rank interventions based on their relative effectiveness. Potential publication bias will be assessed using comparison-adjusted funnel plots. Additionally, a paired random-effects meta-analysis will compare the efficacy of various lower limb strengthening exercise modalities against the control group. Heterogeneity in pairwise comparisons will be assessed using the I^2^ statistic, while publication bias will be evaluated using the Egger test *p*-value. All analyses will be conducted using Stata 15.0 software.

## 3 Results

### 3.1 Study selection

**Figure 1:**
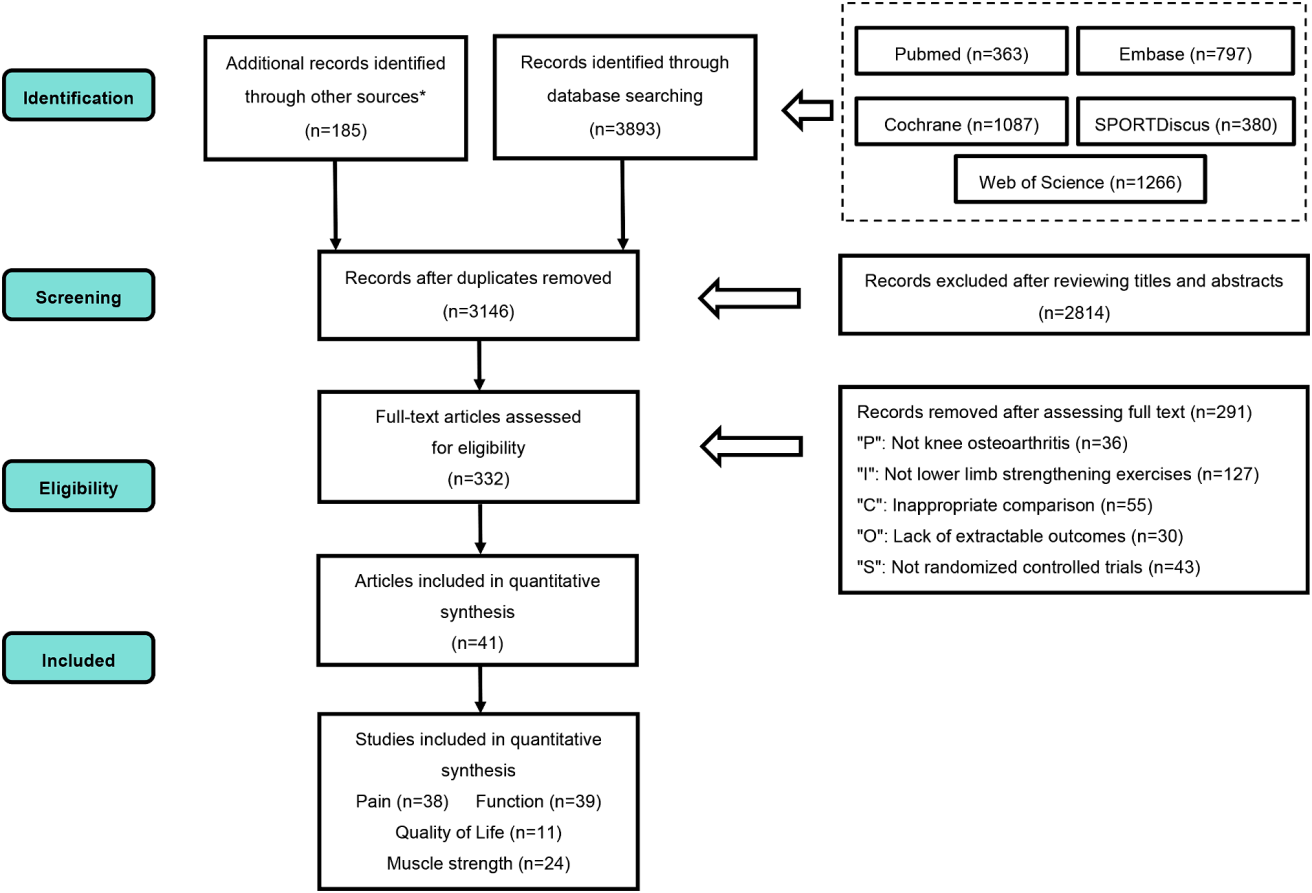
PRISMA flow diagram. PRISMA, preferred reporting items for systematic review and meta-analysis; P, population; I, intervention; C, comparison; O, outcomes; S, study design. * Additional records were obtained by reviewing the reference lists of 11 systematic reviews published within the last five years.

A total of 4,078 records were retrieved, including 185 obtained from the reference lists of 11 systematic reviews (11, 12, 35–43) published in the past 5 years. After duplicate removal, 3,146 unique records remained. Title and abstract screening led to the exclusion of 2,814 records. Full-text review resulted in the inclusion of 41 studies (25, 26, 44–82) in the NMA (Figure 1).

### 3.2 Study characteristics

The analysis encompassed 41 studies, totaling 2,251 patients (1,387 in experimental groups, 864 in control groups). The studies focused on various exercise types: isotonic (*n* = 21), isometric (*n* = 11), isokinetic (*n* = 13), and mixed strengthening (*n* = 8). Professional guidance and supervision were reported in 39 studies. The included trials reported various outcomes of interest: pain-related (*n* = 38), functional improvement (*n* = 39), quality of life (*n* = 11), and muscle strength changes (*n* = 24). Supplementary Appendix 3 presents detailed study characteristics. Details of the exercise prescriptions involved in the included studies are presented in Supplementary Appendix 10.

### 3.3 Quality assessment of included studies

**Figure 2:**
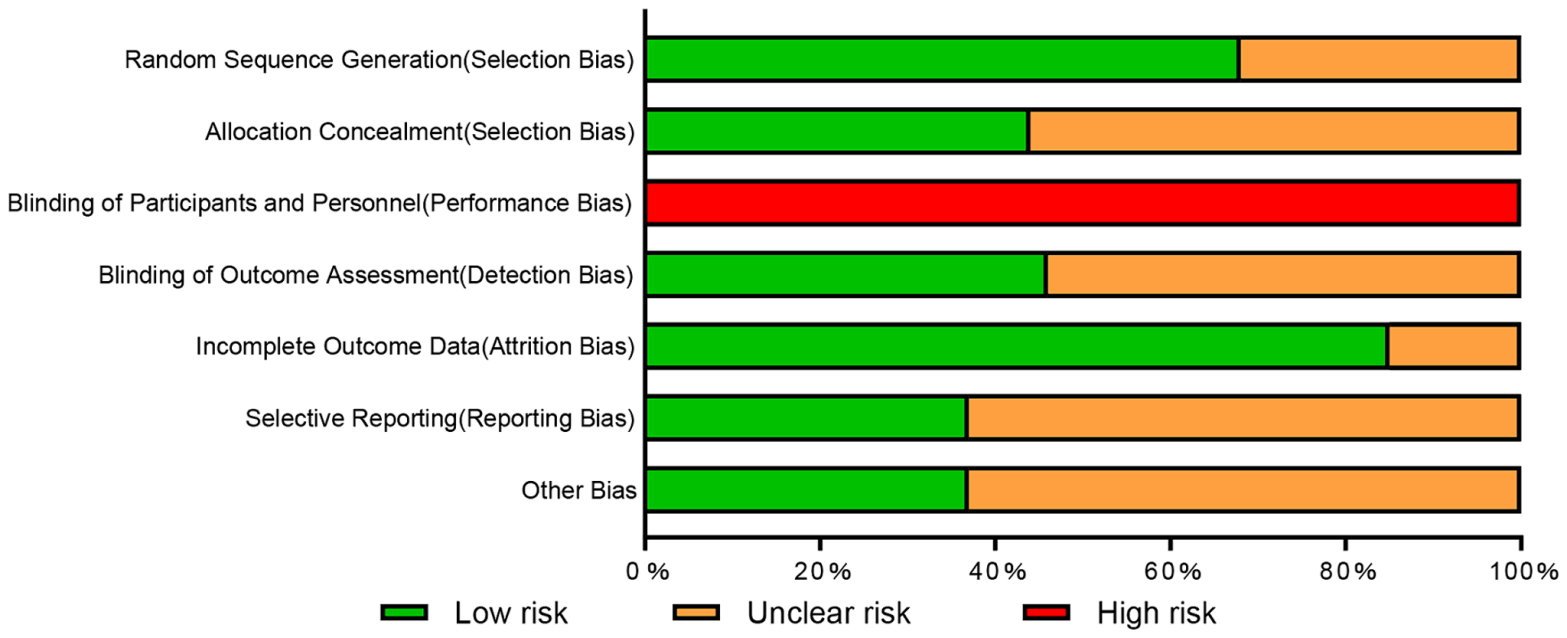
Risk of bias graph.

Twenty-eight studies employed rigorous randomization methods to reduce selection bias, while 13 did not report specific randomization procedures. Allocation concealment methods were reported in 18 studies. The nature of strengthening exercises made participant blinding challenging. Consequently, all 41 studies were assessed as having high risk of performance bias. Outcome assessor blinding was reported in 19 studies. Thirty-five studies demonstrated good outcome data integrity. Pre-registration, which reduces selective reporting risk, was conducted in 15 studies. Supplementary Appendix 4 presents individual study risk of bias assessments, while Figure 2 provides a summary.

### 3.4 Evaluation of intervention efficiency

#### 3.4.1 Pain

**Figure 3:**
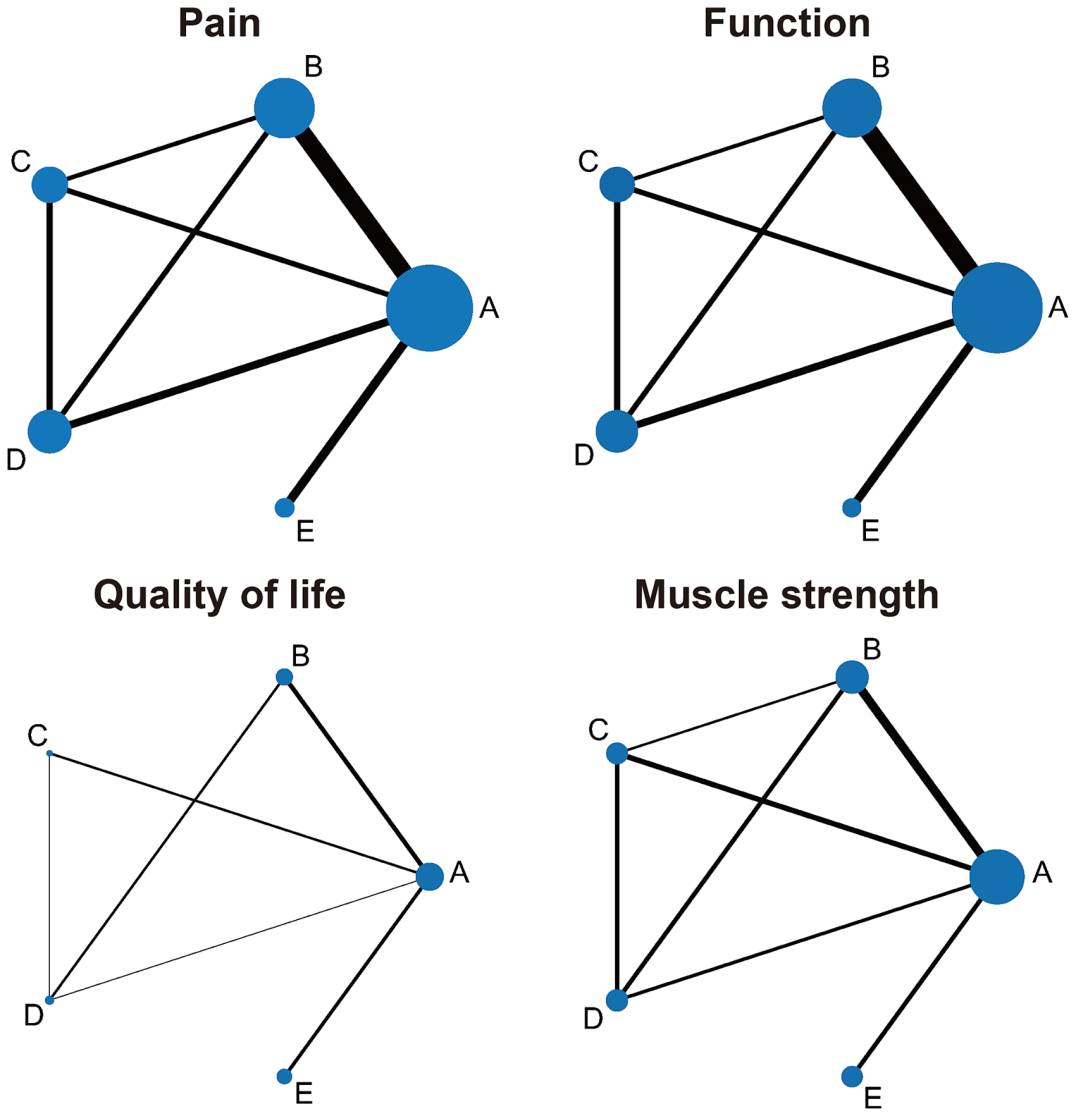
Network evidence map of lower limb strengthening exercises based on different muscle contraction characteristics for knee osteoarthritis. A, control group; B, isotonic exercise; C, isometric exercise; D, isokinetic exercise; E, mixed strengthening exercise. The size of the nodes relates to the number of participants in that intervention type and the thickness of lines between interventions relates to the number of studies for that comparison.

**Figure 4:**
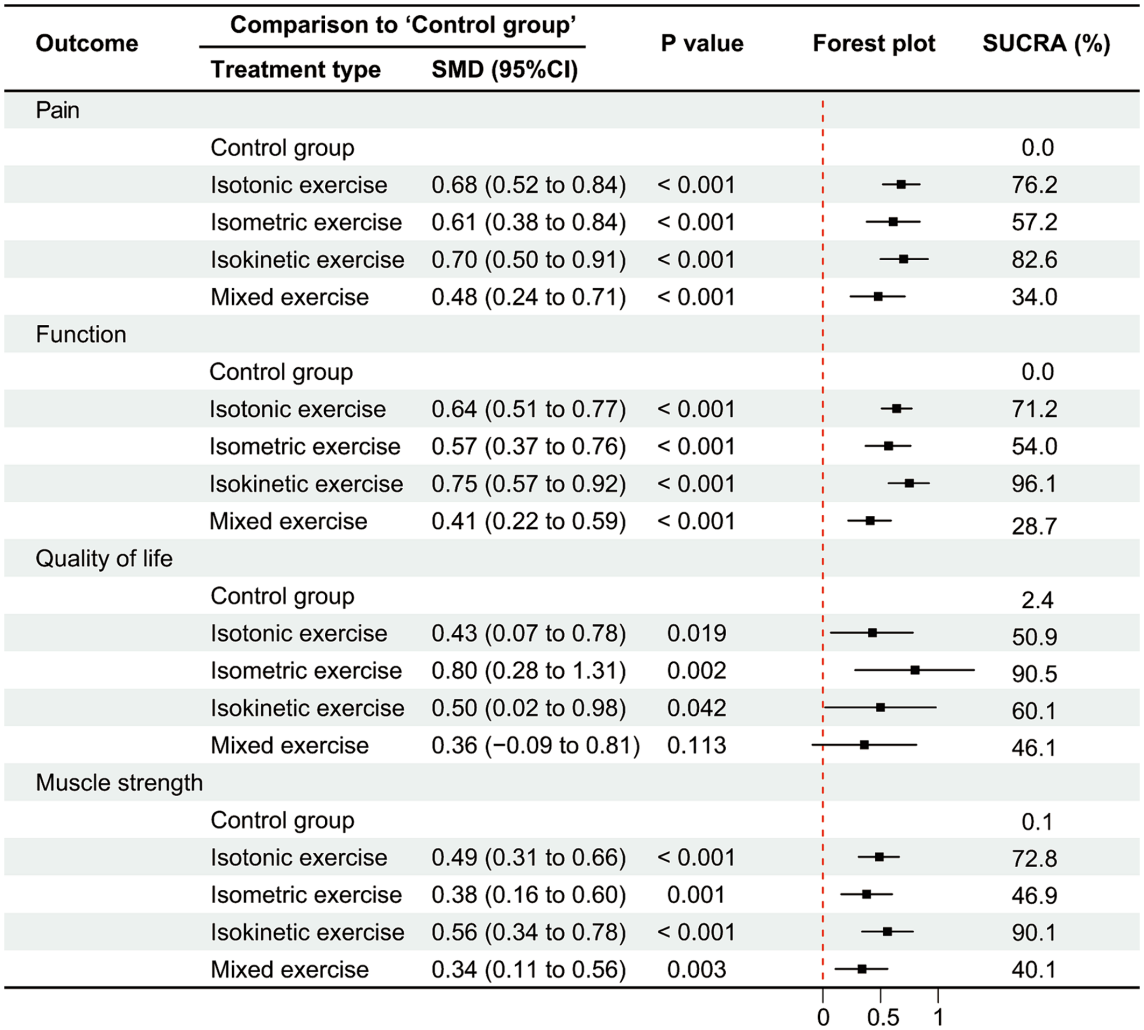
Network meta-analysis results of lower limb strengthening exercises based on different muscle contraction characteristics for knee osteoarthritis mixed exercise, mixed strengthening exercise (The combination of different types of lower limb muscle contraction methods in strengthening exercises).

Thirty-eight studies evaluated pain outcomes, with the network evidence map (Figure 3) illustrating treatment comparisons. The network’s global inconsistency test demonstrated good agreement (χ^2^ = 13.47, *p* = 0.26), with the node-splitting method further corroborating the local consistency (Supplementary Appendix 5). Consistency-model-based NMA results revealed significant pain relief compared to the control group for isotonic exercise (SMD = 0.68, 95% CI: 0.52–0.84), isometric exercise (SMD = 0.61, 95% CI: 0.38–0.84), isokinetic exercise (SMD = 0.70, 95% CI: 0.50–0.91), and mixed strengthening exercise (SMD = 0.48, 95% CI: 0.24–0.71). Isokinetic exercise emerged as the most effective intervention for pain reduction (SUCRA = 82.6%), while mixed strengthening exercise ranked lowest among the four modalities (SUCRA = 34.0%). Figure 4 and Supplementary Appendix 6 present detailed results. No apparent publication bias was detected on the funnel plot (Supplementary Appendix 7). Paired meta-analyses using the random-effects model demonstrated superior efficacy of all strengthening exercise types compared to the control group (Supplementary Appendix 8).

To examine the influence of intervention frequency on primary outcomes, interventions were categorized as low-frequency (≤3 times/week) or high-frequency (≥5 times/week). NMA results indicated that high-frequency interventions significantly outperformed low-frequency interventions for isotonic, isometric, and isokinetic exercise. No statistically significant difference was observed between frequency groups for mixed strengthening exercise. High-frequency isokinetic exercise demonstrated the highest efficacy ranking (SUCRA = 89.9%), while low-frequency mixed strengthening exercise ranked lowest (SUCRA = 25.5%). Supplementary Appendix 9 provides comprehensive results.

#### 3.4.2 Function

Thirty-nine studies evaluated physical function, with the network evidence map (Figure 3) illustrating treatment comparisons. The network’s global inconsistency test demonstrated good agreement (χ^2^ = 8.09, *p* = 0.70), with the node-splitting method corroborating the local consistency (Supplementary Appendix 5). Consistency-model-based NMA results revealed significant functional improvements compared to the control group for isotonic exercise (SMD = 0.64, 95% CI: 0.51–0.77), isometric exercise (SMD = 0.57, 95% CI: 0.37–0.76), isokinetic exercise (SMD = 0.75, 95% CI: 0.57–0.92), and mixed strengthening exercise (SMD = 0.41, 95% CI: 0.22–0.59). Isokinetic exercise emerged as the most effective intervention for functional improvement (SUCRA = 96.1%), while mixed strengthening exercise ranked lowest among the four modalities (SUCRA = 28.7%). Figure 4 and Supplementary Appendix 6 present detailed results. No apparent publication bias was detected on the funnel plot (Supplementary Appendix 7). Paired meta-analyses using the random-effects model demonstrated superior efficacy of all strengthening exercise types compared to the control group (Supplementary Appendix 8).

#### 3.4.3 Quality of life

Eleven studies evaluated quality of life outcomes, with the network evidence map (Figure 3) illustrating treatment comparisons. The network’s global inconsistency test demonstrated good agreement (χ^2^ = 2.33, *p* = 0.31), with the node-splitting method corroborating the local consistency (Supplementary Appendix 5). Consistency-model-based NMA results revealed significant quality of life improvements compared to the control group for isotonic exercise (SMD = 0.43, 95% CI: 0.07–0.78), isometric exercise (SMD = 0.80, 95% CI: 0.28–1.31), and isokinetic exercise (SMD = 0.50, 95% CI: 0.02–0.98). Mixed strengthening exercise, however, did not demonstrate statistically significant differences compared to the control group (SMD = 0.36, 95% CI: −0.09–0.81). Isometric exercise emerged as the most effective intervention for improving quality of life (SUCRA = 90.5%). Figure 4 and Supplementary Appendix 6 present detailed results. No apparent publication bias was detected on the funnel plot (Supplementary Appendix 7). Paired meta-analyses using the random-effects model demonstrated that isometric and isokinetic exercises significantly improved quality of life compared to the control group, while isotonic and mixed strengthening exercise showed no statistically significant differences (Supplementary Appendix 8).

#### 3.4.4 Muscle strength

Twenty-four studies evaluated muscle strength outcomes, with the network evidence map (Figure 3) illustrating treatment comparisons. The network’s global inconsistency test demonstrated good agreement (χ^2^ = 2.55, *p* = 0.86), with the node-splitting method corroborating the local consistency (Supplementary Appendix 5). Consistency-model-based NMA results revealed significant muscle strength improvements compared to the control group for isotonic exercise (SMD = 0.49, 95% CI: 0.31–0.66), isometric exercise (SMD = 0.38, 95% CI: 0.16–0.60), isokinetic exercise (SMD = 0.56, 95% CI: 0.34–0.78), and mixed strengthening exercise (SMD = 0.34, 95% CI: 0.11–0.56). Isokinetic exercise emerged as the most effective intervention for enhancing muscle strength (SUCRA = 90.1%), while mixed strengthening exercise ranked lowest among the four modalities (SUCRA = 40.1%). Figure 4 and Supplementary Appendix 6 present detailed results. No apparent publication bias was detected on the funnel plot (Supplementary Appendix 7). Paired meta-analyses using the random-effects model demonstrated superior efficacy of all strengthening exercise types compared to the control group (Supplementary Appendix 8).

## 4 Discussion

This NMA encompassed 41 studies, involving 2,251 participants, and evaluated the efficacy of isotonic, isometric, isokinetic, and mixed strengthening exercise on pain, physical function, quality of life, and muscle strength in KOA patients. The findings indicated that isokinetic exercise may be the most effective in alleviating pain, improving function, and enhancing muscle strength. Isometric exercise demonstrated the most significant impact on quality of life. Mixed strengthening exercise consistently ranked lowest in SUCRA scores across all outcomes. Notably, for primary outcome measures, high-frequency interventions for isotonic, isometric, and isokinetic exercise types appeared superior to low-frequency interventions, with high-frequency isokinetic exercise potentially exhibiting the highest efficacy for pain relief. However, no statistically significant difference was observed between high- and low-frequency interventions for mixed strengthening exercise.

### 4.1 Isotonic exercise on KOA

Isotonic exercise ranked second in SUCRA for pain relief, physical function improvement, and muscle strength enhancement, while ranking third for quality of life improvement. Pairwise meta-analysis initially showed no significant quality of life improvement versus control groups, contrasting with the NMA results. This discrepancy likely stems from methodological differences: NMA incorporates both direct and indirect evidence, while pairwise analysis uses only direct comparisons. We used the consistency model for NMA and initially the random-effects model for pairwise analysis. Due to low heterogeneity (I^2^ = 30.4%), we then applied the fixed-effects model for pairwise comparison, which showed that isotonic exercise significantly improved quality of life versus control groups (SMD = 0.32, 95% CI: 0.04 to 0.61), aligning with NMA results.

Despite ranking lower than isokinetic exercise in SUCRA for all outcomes, isotonic exercise is more accessible, using simple, cost-effective equipment easily implemented at home and in communities, enhancing its clinical applicability. Isotonic exercise stimulates muscle-tendon elasticity and neuromuscular activation, improving strength, performance, and adaptability (83). Isotonic exercise encompasses both concentric (muscle shortening) and eccentric (muscle lengthening) contraction modes. Both of these contraction modes have demonstrated effectiveness in alleviating the symptoms of KOA (84). The majority of isotonic exercise studies included in our NMA employed the combination of these two contraction modes. However, current research indicates that concentric and eccentric contraction modes offer distinct advantages. Concentric exercise may be more effective in reducing acute cardiovascular stress in KOA patients compared to eccentric exercise (85). Eccentric exercise may offer superior neural adaptations, potentially improving muscle strength and mass more effectively (86, 87). However, eccentric exercise carries a higher risk of muscle damage and therefore requires careful monitoring (88). In clinical practice, when using isotonic exercise to treat patients with KOA, it’s important to tailor the intensity of the concentric or eccentric contraction modes based on the individual patient’s condition. While ensuring safety, this personalized approach may help optimize the therapeutic effects.

### 4.2 Isometric exercise on KOA

Isometric exercise demonstrated the optimal intervention effect in improving quality of life, ranking first in SUCRA. This significant advantage may be attributed to its low-impact nature. Isometric exercise is considered a safe and effective method for rehabilitation and low-impact training due to its gentle effect on joints, controlled force application, and low metabolic cost (89). As the joint remains stationary during isometric exercise, it minimizes joint stress and pain to the greatest extent possible. Research indicates that isometric exercise significantly reduces levels of inflammatory cytokines, including interleukin-6 (IL-6), tumor necrosis factor-alpha (TNF-*α*), C-reactive protein (CRP), and resistin (RSTN) in KOA patients, demonstrating its efficacy in mitigating inflammatory responses (70). Additionally, this form of exercise enhances the molecular weight and viscosity of hyaluronic acid (HA) in synovial fluid, improving joint lubrication and pain relief. It also effectively protects articular cartilage and modulates inflammation by optimizing the concentration of chondroitin sulfate (CS) in synovial fluid along with adjusting pH levels (90). Katayama et al. found that isometric exercise significantly increases the stiffness of the infrapatellar fat pad and reduces levels of oxygenated and deoxygenated hemoglobin in tissues, thereby improving blood circulation and tissue oxygenation in KOA patients (91). This exercise modality also helps restore neuromuscular function in KOA patients, further enhancing their dynamic and static balance capabilities (92, 93). In summary, although isometric exercise may not be as effective as isotonic and isokinetic exercise in alleviating pain, improving function, and enhancing muscle strength, it demonstrates significant advantages in improving patients’ quality of life. These benefits, coupled with its high safety profile, render isometric exercise a valuable therapeutic option for KOA treatment.

### 4.3 Isokinetic exercise on KOA

Isokinetic exercise appears to be the most effective lower limb strengthening method for treating KOA. It ranks highest in SUCRA for pain relief, physical function improvement, and muscle strength enhancement, while ranking second in improving quality of life. Isokinetic exercise is a form of strengthening exercise that utilizes specialized equipment to maintain a constant speed with joint movement throughout the entire range of motion. A meta-analysis by Coudeyre et al. demonstrated that isokinetic training significantly reduces pain and improves function in patients with KOA, corroborating the findings with our NMA (37). Bahşi et al. reported that isokinetic exercise demonstrated a greater advantage in increasing cartilage thickness compared to isometric and isotonic exercise (44). Research by Malas et al. revealed that isokinetic exercise significantly increases knee extension strength and enhances muscle thickness and fiber length bilaterally, including the contralateral side. In contrast, isotonic exercise only increased bilateral muscle thickness (66). Nambi et al.’s study showed that while isokinetic training did not significantly alter levels of bone morphogenetic proteins (BMPs 2, 4, 6, and 7), it significantly reduced pro-inflammatory cytokines (including C-reactive protein, interleukin-6, and tumor necrosis factor-alpha) (94). This suggests that isokinetic training may have a regulatory effect on the inflammatory response, indicating potential anti-inflammatory benefits. Despite the high efficacy of isokinetic exercise in treating KOA, the cost and limited accessibility of the specialized equipment may restrict its widespread application.

### 4.4 Mixed strengthening exercise on KOA

The efficacy of mixed strengthening exercise fell below our expectations, with its SUCRA ranking lowest across all outcome measures. Upon reviewing relevant literature, we observed that instances where mixed exercise programs demonstrate lower effectiveness compared to single-type exercise interventions were not uncommon in KOA exercise therapy research (11, 95–97). Several factors may contribute to this outcome. First, our NMA is constrained by methodological limitations, including a paucity of direct comparative studies between mixed strengthening exercise and single-type strengthening exercise, which may introduce certain biases in the results. Second, an interference effect may occur, as different types of training stimuli can elicit varied molecular responses, potentially leading to mutual inhibition of training outcomes (98). This effect may also manifest when engaging in mixed strengthening exercise that involve diverse muscle contraction methods. Third, insufficient stimulation is also a concern for specific interventions (99). Mixed strengthening exercise includes various forms of exercise within a given time frame. This approach may result in a reduction in the intensity, duration, or frequency of each individual intervention. If the mixed program does not provide sufficient exercise dosage for each type of strengthening exercise, the specific benefits of each type may be diminished. However, increasing the intensity or frequency of each exercise involved may exceed the patient’s tolerance. Fourth, adherence issues – The complexity of the exercise may affect adherence to exercise therapy (100). Mixed strengthening exercise programs are more complex than single-type plans, which may lead to reduced adherence and affect their effectiveness. These potential explanations are not mutually exclusive and may interact in complex ways. Future studies should focus on elucidating the intricate relationships among these factors and exploring optimal mixed strengthening exercise programs for KOA treatment.

### 4.5 Dose–response

The efficacy of strengthening exercises for patients with KOA is largely contingent upon the exercise prescription design, with the dose–response relationship potentially playing a pivotal role (101). Our NMA demonstrates that high-frequency interventions (≥5 times/week) for strengthening exercises are superior in pain alleviation compared to low-frequency interventions (≤3 times/week), corroborating the findings of Juhl et al. (95). However, this relationship extends beyond frequency, encompassing intensity and duration as well. The meta-analysis by Hua et al. revealed that while both high-intensity and low-intensity strengthening exercises yield similar effects on KOA symptom improvement, high-intensity training significantly enhances knee joint strength (39). Marriott et al. discovered that exercise interventions lasting 3 to 6 months are significantly more effective in ameliorating pain and physical function than those lasting less than 3 months. Interestingly, these improvements did not exhibit a clear correlation with exercise volume or patient adherence (102). These findings underscore the complexity of the dose–response relationship in strengthening exercises for KOA. Further investigation into this relationship is imperative. A more comprehensive examination of the dose–response relationship is needed, considering frequency, intensity, duration, and their interactions. This constitutes the focus of our team’s future research endeavors.

### 4.6 Clinical considerations

When prescribing strengthening exercises for patients with KOA, it is imperative to meticulously consider each patient’s pain levels and overall condition. The intensity and modality of exercise should be judiciously calibrated. For instance, during periods of exacerbated pain, low-impact exercises such as isometric training may be particularly beneficial for patients. This form of exercise minimizes joint movement and stress, thereby mitigating the risk of condition aggravation (103). To regulate exercise intensity, a progressive approach is recommended. This strategy may involve initiating with low-intensity exercises and gradually escalating the intensity as the patient’s tolerance improves. Vigilant monitoring of pain during and after exercise is crucial. If pain significantly intensifies during exercise, it may necessitate a reduction in intensity or modification of the exercise regimen. Proper guidance and supervision are of paramount importance, particularly in the initial stages. These facilitate safe and effective exercise performance by patients (10).

### 4.7 Limitations

This study exhibits several limitations. Primarily, the included studies demonstrate varying degrees of bias risk. This potentially compromises the reliability of our findings. Secondly, there is heterogeneity in research methodologies, encompassing disparities in sample sizes, intervention durations, intensities, and control group configurations. This heterogeneity may impede the accurate assessment of intervention effects and outcomes. Thirdly, the prevalence of short follow-up periods limits our ability to evaluate long-term effects. Fourthly, there is an absence of stratified analyses based on factors such as age, gender, or disease severity. This potentially limits the applicability of results to specific patient cohorts. Fifthly, while we conducted analyses on high and low-frequency interventions, we lack comprehensive dose–response analyses, particularly detailed evaluations of exercise intensity and duration. Lastly, the limited number of studies on certain interventions, especially mixed strengthening exercise, restricts comprehensive evaluation.

To address these limitations, future research should prioritize high-quality, well-designed RCTs with standardized protocols, larger sample sizes, and extended follow-up periods. Stratified analyses based on patient characteristics and comprehensive dose–response analyses are imperative to determine optimal exercise parameters. Furthermore, studies should further explore the potential efficacy of mixed strengthening exercise. Research in these areas will yield more robust and clinically relevant evidence for the application of strengthening exercises in KOA treatment.

## 5 Conclusion

This NMA provides comprehensive evidence on the efficacy of lower limb strengthening exercises based on different muscle contraction characteristics for KOA treatment. Our findings suggest that isokinetic exercise may be the most effective in alleviating pain, enhancing function, and improving muscle strength. Isometric exercise demonstrates the most significant impact on quality of life. High-frequency interventions showed superior outcomes compared to low-frequency ones. However, mixed strengthening exercise consistently ranked lowest across all outcomes, warranting further investigation. For the clinical setting, these results support a personalized approach to KOA treatment. While isokinetic exercise shows the highest efficacy, its limited accessibility may favor isotonic or isometric exercise in many situations. Clinicians should consider patient-specific factors such as pain levels, functional status, and available resources when prescribing exercises. These findings should be interpreted with caution due to limitations such as varying degrees of bias risk in included studies and heterogeneity in research methodologies. Future research should focus on high-quality, long-term studies with standardized protocols. These studies should explore optimal mixed strengthening exercise programs and conduct comprehensive dose–response analyses to further refine KOA treatment strategies and improve patient care.

## Data Availability

The original contributions presented in the study are included in the article/Supplementary material, further inquiries can be directed to the corresponding authors.
