# Supplementary material for: Efficacy of lower limb strengthening exercises based on different muscle contraction characteristics for knee osteoarthritis: a systematic review and network meta-analysis
Source: Front Med (Lausanne). 2024 Sep 25;11:1442683. doi: 10.3389/fmed.2024.1442683 (PMC11461219; doi:10.3389/fmed.2024.1442683)
Supplement: Supplementary file 1 [file Data_Sheet_1.PDF]

Table of contents

Appendix 1: Search strategy..2
Appendix 2: Types of lower limb strengthening exercises based on different muscle contraction characteristics..9
Appendix 3: Characteristics of included studies..10
Appendix 4: Risk of bias of included studies..17
Appendix 5: Evaluation of inconsistency 19
Appendix 6: League table and SUCRA 21
Appendix 7: Funnel plots..29
Appendix 8: Results of paired comparison meta-analysis...31
Appendix 9: Results under different intervention frequencies for pain.......................32
Appendix 10: Specific details of the exercise prescription included in the study........36

Appendix 1: Search strategy

**Comprehensive search**		
**Pubmed**		
#1	("Osteoarthritis, Knee"[Mesh]) OR (((((Osteoarthritis, Knee[Title/Abstract]) OR (Knee Osteoarthritides[Title/Abstract])) OR (Knee Osteoarthritis[Title/Abstract])) OR (Osteoarthritis of Knee[Title/Abstract])) OR (Osteoarthritis of the Knee[Title/Abstract]))	36471
#2	("Resistance Training"[Mesh]) OR (Resistance Training[Title/Abstract]) OR (Training, Resistance[Title/Abstract]) OR (Strength Training[Title/Abstract]) OR (Training, Strength[Title/Abstract]) OR (Weight-Lifting Strengthening Program*[Title/Abstract]) OR (Strengthening Program*, Weight-Lifting[Title/Abstract]) OR (Weight Lifting Strengthening Program*[Title/Abstract]) OR (Weight-Lifting Exercise Program*[Title/Abstract]) OR (Exercise Program*, Weight-Lifting[Title/Abstract]) OR (Weight Lifting Exercise Program*[Title/Abstract]) OR (Weight-Bearing Strengthening Program*[Title/Abstract]) OR (Strengthening Program*, Weight-Bearing[Title/Abstract]) OR (Weight Bearing Strengthening Program*[Title/Abstract]) OR (Weight-Bearing Exercise Program*[Title/Abstract]) OR (Exercise Program*, Weight-Bearing[Title/Abstract]) OR (Weight Bearing Exercise Program*[Title/Abstract]) OR (Strengthening exercise*[Title/Abstract]) OR (Strengthening training[Title/Abstract])	25840
#3	("Isometric Contraction"[Mesh]) OR (Isometric Contraction*[Title/Abstract]) OR (Contraction*, Isometric[Title/Abstract]) OR (isometric training[Title/Abstract]) OR (isometric exercise*[Title/Abstract]) OR (isokinetic training[Title/Abstract]) OR (isokinetic exercise*[Title/Abstract]) OR (isotonic training[Title/Abstract]) OR (isotonic exercise*[Title/Abstract]) OR (isotonic contraction*[Title/Abstract]) OR (contraction* isotonic[Title/Abstract]) OR (constant speed exercise*[Title/Abstract]) OR (concentric exercise*[Title/Abstract]) OR (concentric contraction*[Title/Abstract]) OR (contraction* concentric[Title/Abstract]) OR (eccentric exercise*[Title/Abstract]) OR (eccentric contraction*[Title/Abstract]) OR (contraction* eccentric[Title/Abstract])	28569
#4	(band[Title/Abstract]) OR (squat[Title/Abstract]) OR (Plank[Title/Abstract]) OR (Press[Title/Abstract]) OR (weightlifting[Title/Abstract]) OR (dumbbell[Title/Abstract]) OR (pull-up[Title/Abstract]) OR (Deadlift[Title/Abstract])	261655
#5	#2 OR #3 OR #4	308362
#6	randomized controlled trial[Publication Type] OR randomized[Title/Abstract] OR placebo[Title/Abstract]	1078228
#7	#1 AND #5 AND #6	363
**Embase**		

#1	'knee osteoarthritis'/exp OR (arthrosis NEAR/2 knee):ab,ti OR 'femorotibial arthrosis':ab,ti OR gonarthrosis:ab,ti OR (knee NEAR/2 arthrosis):ab,ti OR (knee NEAR/2 joint NEAR/2 arthrosis):ab,ti OR (knee NEAR/2 joint NEAR/2 osteoarthritis):ab,ti OR (knee NEAR/2 osteo-arthritis):ab,ti OR (knee NEAR/2 osteo-arthrosis):ab,ti OR (knee NEAR/2 osteoarthrosis):ab,ti OR (osteoarthritis NEAR/2 knee):ab,ti OR (osteoarthrosis NEAR/2 knee):ab,ti OR 'knee osteoarthritis':ab,ti	52225
#2	'resistance training'/exp OR 'resistance training':ab,ti OR 'training, resistance':ab,ti OR 'strength training':ab,ti OR 'training, strength':ab,ti OR ((weight NEAR/2 lift* NEAR/2 strengthen* NEAR/2 program*):ab,ti) OR ((weight NEAR/2 lift* NEAR/2 exercise NEAR/2 program*):ab,ti) OR ((weight NEAR/2 bear* NEAR/2 strengthen* NEAR/2 program*):ab,ti) OR ((weight NEAR/2 bear* NEAR/2 exercise NEAR/2 program*):ab,ti) OR 'strengthening exercise*':ab,ti OR 'strengthening training':ab,ti	40080
#3	'isometric contraction'/exp OR 'isometric exercise'/exp OR 'muscle isometric contraction'/exp OR 'isotonic exercise'/exp OR 'isokinetic exercise'/exp OR ((isometric NEAR/2 (contraction* OR training OR exercise* OR endurance OR 'endurance test' OR response)):ab,ti) OR ((contraction* NEAR/2 isometric):ab,ti) OR ((muscle NEAR/2 isometric NEAR/2 (contraction OR tension)):ab,ti) OR ((muscular NEAR/2 isometric NEAR/2 contraction):ab,ti) OR ((isokinetic NEAR/2 (training OR exercise* OR 'muscle contraction')):ab,ti) OR ((isotonic NEAR/2 (training OR exercise* OR contraction*)):ab,ti) OR (('constant speed' NEAR/2 exercise*):ab,ti) OR ((concentric NEAR/2 (exercise* OR contraction*)):ab,ti) OR ((eccentric NEAR/2 (exercise* OR contraction*)):ab,ti)	38597
#4	band:ab,ti OR squat:ab,ti OR plank:ab,ti OR press:ab,ti OR weightlifting:ab,ti OR dumbbell:ab,ti OR 'pull up':ab,ti OR deadlift:ab,ti	459643
#5	#2 OR #3 OR #4	528363
#6	'randomized controlled trial'/exp OR 'controlled clinical trial'/exp OR 'clinical trial'/exp OR randomized:ab,ti OR placebo:ab,ti OR randomly:ab,ti OR trial:ti OR blind*:ab,ti	3091511
#7	#1 AND #5 AND #6	797
**Cochrane Central Register of Controlled Trials (CENTRAL)**		
#1	[mh "Osteoarthritis, Knee"] OR ((Osteoarthritis NEAR/2 Knee):ti,ab,kw) OR ("Knee Osteoarthritides":ti,ab,kw) OR ("Knee Osteoarthritis":ti,ab,kw) OR ("Osteoarthritis of Knee":ti,ab,kw) OR ("Osteoarthritis of the Knee":ti,ab,kw)	14888
#2	[mh "Resistance Training"] OR ("Resistance Training":ti,ab,kw) OR ("Training, Resistance":ti,ab,kw) OR ("Strength Training":ti,ab,kw) OR ("Training, Strength":ti,ab,kw) OR ((Weight-Lifting NEAR/2 Strengthening NEAR/2 (Program NEXT (s OR ing))):ti,ab,kw) OR ((Weight Lifting NEAR/2 Strengthening NEAR/2 (Program NEXT (s OR ing))):ti,ab,kw) OR ((Weight-Lifting NEAR/2 Exercise NEAR/2 (Program NEXT (s OR ing))):ti,ab,kw) OR ((Weight Lifting NEAR/2 Exercise NEAR/2 (Program NEXT (s OR ing))):ti,ab,kw) OR ((Weight-Bearing	18953

	NEAR/2 Strengthening NEAR/2 (Program NEXT (s OR ing))):ti,ab,kw) OR ((Weight Bearing NEAR/2 Strengthening NEAR/2 (Program NEXT (s OR ing))):ti,ab,kw) OR ((Weight-Bearing NEAR/2 Exercise NEAR/2 (Program NEXT (s OR ing))):ti,ab,kw) OR ((Weight Bearing NEAR/2 Exercise NEAR/2 (Program NEXT (s OR ing))):ti,ab,kw) OR ((Strengthening NEXT (exercise OR exercises)):ti,ab,kw) OR ("Strengthening training":ti,ab,kw)	
#3	((Isometric NEXT (Contraction OR Contractions)):ti,ab,kw) OR ((Contraction NEXT Isometric):ti,ab,kw) OR ("isometric training":ti,ab,kw) OR ((isometric NEXT (exercise OR exercises)):ti,ab,kw) OR ("isokinetic training":ti,ab,kw) OR ((isokinetic NEXT (exercise OR exercises)):ti,ab,kw) OR ("isotonic training":ti,ab,kw) OR ((isotonic NEXT (exercise OR exercises)):ti,ab,kw) OR ((isotonic NEXT (contraction OR contractions)):ti,ab,kw) OR ((contraction NEXT isotonic):ti,ab,kw) OR ((constant NEXT speed NEXT (exercise OR exercises)):ti,ab,kw) OR ((concentric NEXT (exercise OR exercises)):ti,ab,kw) OR ((concentric NEXT (contraction OR contractions)):ti,ab,kw) OR ((contraction NEXT concentric):ti,ab,kw) OR ((eccentric NEXT (exercise OR exercises)):ti,ab,kw) OR ((eccentric NEXT (contraction OR contractions)):ti,ab,kw) OR ((contraction NEXT eccentric):ti,ab,kw)	6056
#4	band:ti,ab,kw OR squat:ti,ab,kw OR Plank:ti,ab,kw OR Press:ti,ab,kw OR weightlifting:ti,ab,kw OR dumbbell:ti,ab,kw OR pull-up:ti,ab,kw OR Deadlift:ti,ab,kw	15311
#5	#2 OR #3 OR #4	35493
#6	#1 AND #5	1087
**Web of Science**		
#1	TS=(Osteoarthritis, Knee) OR TS=(Knee Osteoarthritides) OR TS=(Knee Osteoarthritis) OR TS=(Osteoarthritis of Knee) OR TS=(Osteoarthritis of the Knee)	99266
#2	TS=(Resistance Training) OR TS=(Training, Resistance) OR TS=(Strength Training) OR TS=(Training, Strength) OR TS=(Weight-Lifting Strengthening Program*) OR TS=(Strengthening Program*, Weight-Lifting) OR TS=(Weight Lifting Strengthening Program*) OR TS=(Weight-Lifting Exercise Program*) OR TS=(Exercise Program*, Weight-Lifting) OR TS=(Weight Lifting Exercise Program*) OR TS=(Weight-Bearing Strengthening Program*) OR TS=(Strengthening Program*, Weight-Bearing) OR TS=(Weight Bearing Strengthening Program*) OR TS=(Weight-Bearing Exercise Program*) OR TS=(Exercise Program*, Weight-Bearing) OR TS=(Weight Bearing Exercise Program*) OR TS=(Strengthening exercise*) OR TS=(Strengthening training)	330546
#3	TS=(Isometric Contraction*) OR TS=(Contraction*, Isometric) OR TS=(isometric training) OR TS=(isometric exercise*) OR TS=(isokinetic training) OR TS=(isokinetic exercise*) OR TS=(isotonic training) OR TS=(isotonic exercise*) OR TS=(isotonic contraction*) OR TS=(contraction*	81310

	isotonic) OR TS=(constant speed exercise*) OR TS=(concentric exercise*) OR TS=(concentric contraction*) OR TS=(contraction* concentric) OR TS=(eccentric exercise*) OR TS=(eccentric contraction*) OR TS=(contraction* eccentric)	
#4	TS=(band) OR TS=(squat) OR TS=(Plank) OR TS=(Press) OR TS=(weightlifting) OR TS=(dumbbell) OR TS=(pull-up) OR TS=(Deadlift)	6744660
#5	#2 OR #3 OR #4	7116824
#6	TS=randomized OR TS=placebo	1760728
#7	#1 AND #5 AND #6	1266
**SPORTDiscus**		
#1	(TI Osteoarthritis, Knee OR AB Osteoarthritis, Knee) OR (TI Knee Osteoarthritides OR AB Knee Osteoarthritides) OR (TI Knee Osteoarthritis OR AB Knee Osteoarthritis) OR (TI Osteoarthritis of Knee OR AB Osteoarthritis of Knee) OR (TI Osteoarthritis of the Knee OR AB Osteoarthritis of the Knee)	64568
#2	(TI "Resistance Training" OR AB "Resistance Training") OR (TI "Training, Resistance" OR AB "Training, Resistance") OR (TI "Strength Training" OR AB "Strength Training") OR (TI "Training, Strength" OR AB "Training, Strength") OR (TI "Weight-Lifting Strengthening Program*" OR AB "Weight-Lifting Strengthening Program*") OR (TI "Strengthening Program*, Weight-Lifting" OR AB "Strengthening Program*, Weight-Lifting") OR (TI "Weight Lifting Strengthening Program*" OR AB "Weight Lifting Strengthening Program*") OR (TI "Weight-Lifting Exercise Program*" OR AB "Weight-Lifting Exercise Program*") OR (TI "Exercise Program*, Weight-Lifting" OR AB "Exercise Program*, Weight-Lifting") OR (TI "Weight Lifting Exercise Program*" OR AB "Weight Lifting Exercise Program*") OR (TI "Weight-Bearing Strengthening Program*" OR AB "Weight-Bearing Strengthening Program*") OR (TI "Strengthening Program*, Weight-Bearing" OR AB "Strengthening Program*, Weight-Bearing") OR (TI "Weight Bearing Strengthening Program*" OR AB "Weight Bearing Strengthening Program*") OR (TI "Weight-Bearing Exercise Program*" OR AB "Weight-Bearing Exercise Program*") OR (TI "Exercise Program*, Weight-Bearing" OR AB "Exercise Program*, Weight-Bearing") OR (TI "Weight Bearing Exercise Program*" OR AB "Weight Bearing Exercise Program*") OR (TI "Strengthening exercise*" OR AB "Strengthening exercise*") OR (TI "Strengthening training" OR AB "Strengthening training")	18337
#3	(TI Isometric Contraction* OR AB Isometric Contraction*) OR (TI Contraction*, Isometric OR AB Contraction*, Isometric) OR (TI isometric training OR AB isometric training) OR (TI isometric exercise* OR AB isometric exercise*) OR (TI isokinetic training OR AB isokinetic training) OR (TI isokinetic exercise* OR AB isokinetic exercise*) OR (TI isotonic training OR AB isotonic training) OR (TI isotonic exercise* OR AB isotonic exercise*) OR (TI isotonic contraction* OR AB isotonic contraction*) OR (TI contraction* isotonic OR	15483

	AB contraction* isotonic) OR (TI constant speed exercise* OR AB constant speed exercise*) OR (TI concentric exercise* OR AB concentric exercise*) OR (TI concentric contraction* OR AB concentric contraction*) OR (TI contraction* concentric OR AB contraction* concentric) OR (TI eccentric exercise* OR AB eccentric exercise*) OR (TI eccentric contraction* OR AB eccentric contraction*) OR (TI contraction* eccentric OR AB contraction* eccentric)	
#4	(TI "band" OR AB "band") OR (TI "squat" OR AB "squat") OR (TI "Plank" OR AB "Plank") OR (TI "Press" OR AB "Press") OR (TI "weightlifting" OR AB "weightlifting") OR (TI "dumbbell" OR AB "dumbbell") OR (TI "pull-up" OR AB "pull-up") OR (TI "Deadlift" OR AB "Deadlift")	27024
#5	#2 OR #3 OR #4	54378
#6	#1 AND #5	380

**Relevant systematic reviews and meta-analyses**		
**Pubmed**		
#1	("Osteoarthritis, Knee"[Mesh]) OR (((((Osteoarthritis, Knee[Title/Abstract]) OR (Knee Osteoarthritides[Title/Abstract])) OR (Knee Osteoarthritis[Title/Abstract])) OR (Osteoarthritis of Knee[Title/Abstract])) OR (Osteoarthritis of the Knee[Title/Abstract]))	36471
#2	("Resistance Training"[Mesh]) OR (Resistance Training[Title/Abstract]) OR (Training, Resistance[Title/Abstract]) OR (Strength Training[Title/Abstract]) OR (Training, Strength[Title/Abstract]) OR (Weight-Lifting Strengthening Program*[Title/Abstract]) OR (Strengthening Program*, Weight-Lifting[Title/Abstract]) OR (Weight Lifting Strengthening Program*[Title/Abstract]) OR (Weight-Lifting Exercise Program*[Title/Abstract]) OR (Exercise Program*, Weight-Lifting[Title/Abstract]) OR (Weight Lifting Exercise Program*[Title/Abstract]) OR (Weight-Bearing Strengthening Program*[Title/Abstract]) OR (Strengthening Program*, Weight-Bearing[Title/Abstract]) OR (Weight Bearing Strengthening Program*[Title/Abstract]) OR (Weight-Bearing Exercise Program*[Title/Abstract]) OR (Exercise Program*, Weight-Bearing[Title/Abstract]) OR (Weight Bearing Exercise Program*[Title/Abstract]) OR (Strengthening exercise*[Title/Abstract]) OR (Strengthening training[Title/Abstract])	25840
#3	("Isometric Contraction"[Mesh]) OR (Isometric Contraction*[Title/Abstract]) OR (Contraction*, Isometric[Title/Abstract]) OR (isometric training[Title/Abstract]) OR (isometric exercise*[Title/Abstract]) OR (isokinetic training[Title/Abstract]) OR (isokinetic exercise*[Title/Abstract]) OR (isotonic training[Title/Abstract]) OR (isotonic exercise*[Title/Abstract]) OR (isotonic contraction*[Title/Abstract]) OR (contraction* isotonic[Title/Abstract]) OR (constant speed exercise*[Title/Abstract]) OR (concentric	28569

	exercise*[Title/Abstract]) OR (concentric contraction*[Title/Abstract]) OR (contraction* concentric[Title/Abstract]) OR (eccentric exercise*[Title/Abstract]) OR (eccentric contraction*[Title/Abstract]) OR (contraction* eccentric[Title/Abstract])	
#4	(band[Title/Abstract]) OR (squat[Title/Abstract]) OR (Plank[Title/Abstract]) OR (Press[Title/Abstract]) OR (weightlifting[Title/Abstract]) OR (dumbbell[Title/Abstract]) OR (pull-up[Title/Abstract]) OR (Deadlift[Title/Abstract])	261655
#5	#2 OR #3 OR #4	308362
#6	("systematic review"[Title/Abstract] OR "systematic reviews"[Title/Abstract] OR "meta-analysis"[Title/Abstract] OR "meta-analyses"[Title/Abstract] OR "meta analysis"[Title/Abstract] OR "meta analyses"[Title/Abstract] OR "systematic literature review"[Title/Abstract] OR "systematic literature reviews"[Title/Abstract]) AND ("systematic review"[Publication Type] OR "meta-analysis"[Publication Type]) AND ("2019/01/01"[Date - Publication] : "2024/8/17"[Date - Publication])	175737
#7	#1 AND #5 AND #6	30
**Embase**		
#1	'knee osteoarthritis'/exp OR (arthrosis NEAR/2 knee):ab,ti OR 'femorotibial arthrosis':ab,ti OR gonarthrosis:ab,ti OR (knee NEAR/2 arthrosis):ab,ti OR (knee NEAR/2 joint NEAR/2 arthrosis):ab,ti OR (knee NEAR/2 joint NEAR/2 osteoarthritis):ab,ti OR (knee NEAR/2 osteo-arthritis):ab,ti OR (knee NEAR/2 osteo-arthrosis):ab,ti OR (knee NEAR/2 osteoarthrosis):ab,ti OR (osteoarthritis NEAR/2 knee):ab,ti OR (osteoarthrosis NEAR/2 knee):ab,ti OR 'knee osteoarthritis':ab,ti	52225
#2	'resistance training'/exp OR 'resistance training':ab,ti OR 'training, resistance':ab,ti OR 'strength training':ab,ti OR 'training, strength':ab,ti OR ((weight NEAR/2 lift* NEAR/2 strengthen* NEAR/2 program*):ab,ti) OR ((weight NEAR/2 lift* NEAR/2 exercise NEAR/2 program*):ab,ti) OR ((weight NEAR/2 bear* NEAR/2 strengthen* NEAR/2 program*):ab,ti) OR ((weight NEAR/2 bear* NEAR/2 exercise NEAR/2 program*):ab,ti) OR 'strengthening exercise*':ab,ti OR 'strengthening training':ab,ti	40080
#3	'isometric contraction'/exp OR 'isometric exercise'/exp OR 'muscle isometric contraction'/exp OR 'isotonic exercise'/exp OR 'isokinetic exercise'/exp OR ((isometric NEAR/2 (contraction* OR training OR exercise* OR endurance OR 'endurance test' OR response)):ab,ti) OR ((contraction* NEAR/2 isometric):ab,ti) OR ((muscle NEAR/2 isometric NEAR/2 (contraction OR tension)):ab,ti) OR ((muscular NEAR/2 isometric NEAR/2 contraction):ab,ti) OR ((isokinetic NEAR/2 (training OR exercise* OR 'muscle contraction')):ab,ti) OR ((isotonic NEAR/2 (training OR exercise* OR contraction*)):ab,ti) OR (('constant speed' NEAR/2 exercise*):ab,ti) OR ((concentric NEAR/2 (exercise* OR contraction*)):ab,ti) OR ((eccentric NEAR/2 (exercise* OR contraction*)):ab,ti)	38597
#4	band:ab,ti OR squat:ab,ti OR plank:ab,ti OR press:ab,ti OR weightlifting:ab,ti OR dumbbell:ab,ti OR 'pull up':ab,ti OR deadlift:ab,ti	459643

#5	#2 OR #3 OR #4	528363
#6	('systematic review':ti,ab OR 'systematic reviews':ti,ab OR 'meta-analysis':ti,ab OR 'meta-analyses':ti,ab OR 'meta analysis':ti,ab OR 'meta analyses':ti,ab OR 'systematic literature review':ti,ab OR 'systematic literature reviews':ti,ab) AND ('systematic review'/exp OR 'systematic review' OR 'meta analysis'/exp OR 'meta analysis') AND [2019-2024]/py	306321
#7	#1 AND #5 AND #6	89

Appendix 2: Types of lower limb strengthening exercises based on different muscle contraction characteristics

Type	Definition
Isotonic exercise	Isotonic exercise is a form of strengthening exercise that involves muscle contraction with changes in muscle length. It includes both concentric (muscle shortening) and eccentric (muscle lengthening) contractions. During isotonic exercise, the muscle maintains relatively constant tension while changing length throughout the range of motion (1). For example, leg press and squats.
Isometric exercise	Isometric exercise is a type of strengthening exercise where the muscle contracts and produces force without a change in muscle length or joint angle (1). The muscle generates tension against an immovable resistance or holds a static position. For example, Wall Sit and Isometric Leg Extension.
Isokinetic exercise	Isokinetic exercise is a form of strengthening exercise that involves muscle contractions at a constant speed throughout the entire range of motion. This is achieved using specialized motorized exercise machines, typically isokinetic dynamometers, which ensure that the speed of movement remains constant while allowing the muscles to exert maximal force at every point (2). For example, isokinetic knee flexion and extension on an isokinetic device.
Mixed strengthening exercise	Two or more of the specific types of exercise training mentioned above (not deemed multimodal if only part of warm up or cool down).

References

1. Widodo AF, Tien CW, Chen CW, Lai SC. Isotonic and Isometric Exercise Interventions Improve the Hamstring Muscles' Strength and Flexibility: A Narrative Review. Healthcare (Basel, Switzerland). 2022;10(5).
2. Coudeyre E, Jegu AG, Giustanini M, Marrel JP, Edouard P, Pereira B. Isokinetic muscle strengthening for knee osteoarthritis: A systematic review of randomized controlled trials with meta-analysis. Annals of physical and rehabilitation medicine. 2016;59(3):207-15.

Appendix 3: Characteristics of included studies

#	Study ID	Trial registration	Sample size	Age	Gender (Male/Female)	Treatment group	Frequency (weekly)	Control group	Duration of treatment	Outcomes
1	Bahşi, et al. 2022	-	TG1:20<br>TG2:20<br>TG3:20	TG1:54.6±6.1<br>TG2:56.3±6.6<br>TG3:52.7±5.8	4/56	TG1:Isotonic exercise*<br>TG2:Isometric exercise*<br>TG3:Isokinetic exercise*	5 times	-	3 Weeks	1.VAS<br>2.WOMAC (function)<br>3.Extensor peak torque at 60°/s
2	Baker, et al. 2001	-	TG:23<br>CG:23	TG:69.0±6.0<br>CG:68.0±6.0	10/36	Isotonic exercise*	3 times	Health education	16 Weeks	1.WOMAC (pain)<br>2.WOMAC (function)<br>3.Total knee extension 1RM strength<br>4.SF-36 mental health
3	Bennell, et al. 2010	ACTR12607000001493	TG:45<br>CG:44	TG:64.5±9.1<br>CG:64.6±7.6	46/43	Isotonic exercise +Isometric exercise*	5 times	No intervention	12 Weeks	1.NRS<br>2.WOMAC (function)<br>3.Isometric contraction strength of the quadriceps
4	Bruce-Brand, et al. 2012	ISRCTN85231954	TG:10<br>CG:6	TG:63.4±5.9<br>CG:65.2±3.1	9/7	Isotonic exercise +Isometric exercise*	3 times	Usual care	6 Weeks	1.WOMAC (pain)<br>2.WOMAC (function)<br>3.Extensor peak torque at 60°/s<br>4.SF-36 mental health

5	Çakır, et al. 2016	-	TG1:36<br>TG2:37	TG1:61.0±7.6<br>TG2:64.5±5.4	0/73	TG1:Isometric exercise<br>TG2:Isokinetic exercise*	5 times	-	2 Weeks	1.WOMAC (pain)<br>2.WOMAC (function)<br>3.Extensor peak torque at 90°/s
6	Chang, et al. 2012	-	TG:24<br>CG:17	TG:65.0±8.4<br>CG:70.8±8.4	0/41	Isotonic exercise +Isometric exercise*	3 times	Usual care	8 Weeks	1.WOMAC (pain)<br>2.WOMAC (function)
7	Cheing, et al, 2004	-	TG:17<br>CG:16	TG:64.3+9.2<br>CG:65.3+8.3	6/27	Isometric exercise*	5 times	Usual care	4 Weeks	1.Extensor peak torque at 60°/s
8	DeVita, et al, 2018	NCT01538407	TG:15<br>CG:15	TG:58.1±6.5<br>CG:56.2±8.9	12/18	Isotonic exercise*	3 times	No intervention	12 Weeks	1.WOMAC (pain)<br>2.WOMAC (function)<br>3.Extensor peak torque at 60°/s
9	Doss D, et al. 2014	-	TG:37<br>CG:36	TG:49.9±4.0<br>CG:50.8±4.7	24/49	Isotonic exercise*	5 times	Usual care	4 Weeks	1.WOMAC (pain)<br>2.WOMAC (function)
10	Egwu, et al. 2018	-	TG:37<br>CG:42	TG:50.1±10.2<br>CG:53.9±10.4	13/66	Isotonic exercise +Isometric exercise*	3 times	Health education	6 Weeks	1.VAS<br>2.IKHOAM
11	Eyigor, et al. 2004	-	TG1:18<br>TG2:21	TG1:51.9±8.1<br>TG2:53.1±6.7	6/33	TG1:Isotonic exercise<br>TG2:Isokinetic exercise*	3 times	-	6 Weeks	1.WOMAC (pain)<br>2.WOMAC (function)<br>3.Extensor peak torque

										at 60°/s<br>4.SF-36 mental health
12	Foroughi, et al. 2011	ACTRN12605000116628	TG:26<br>CG:28	TG:66.0±8.0<br>CG:65.0±7.0	0/54	Isotonic exercise*	3 times	No intervention	24 Weeks	1.WOMAC (pain)<br>2.WOMAC (function)<br>3.Total knee extension 1RM strength
13	Gür, et al. 2002	-	TG:8<br>CG:6	TG:55.0±12.0<br>CG:57.0±9.0	-	Isokinetic exercise*	3 times	No intervention	8 Weeks	1.NRS<br>2.Functional Scale
14	Hsu, et al. 2021	NCT03973463	TG:21<br>CG:21	TG:65.6±3.9<br>CG:66.0±3.9	15/27	Isotonic exercise*	3 times	Usual care	12 Weeks	1.WOMAC (pain)<br>2.WOMAC (function)
15	Huang, et al. 2003	-	TG1:33<br>TG2:33<br>TG3:33<br>CG:33	62.0±4.5	39/93	TG1:Isotonic exercise*<br>TG2:Isometric exercise*<br>TG3:Isokinetic exercise*	3 times	Usual care	8 Weeks	1.VAS<br>2.Lequesne Index
16	Huang, et al. 2005	-	TG:35<br>CG:35	TG:65.0±6.4<br>CG:65.0±6.4	13/57	Isokinetic exercise*	3 times	Usual care	8 weeks	1.VAS<br>2.Lequesne Index
17	Imoto, et al. 2012	-	TG:50<br>CG:50	TG:61.5±6.9<br>CG:58.8±9.6	8/94	Isotonic exercise*	2 times	Health education	8 Weeks	1.NRS<br>2.SF-36 functional capacity<br>3.SF-36 mental health
18	Jan, et al. 2008	-	TG:34<br>CG:30	TG:63.3±6.6<br>CG:62.8±6.3	12/52	Isotonic exercise*	3 times	No intervention	8 Weeks	1.WOMAC (pain)<br>2.WOMAC (function)

										3.Extensor peak torque at 60°/s
19	Jan, et al. 2009	NCT9100002377	TG:36<br>CG:35	TG:62.0±6.7<br>CG:62.2±6.7	23/48	Isotonic exercise*	3 times	No intervention	8 Weeks	1.WOMAC (function)<br>2.Extensor peak torque at 60°/s
20	Jorge, et al. 2015	-	TG:29<br>CG:31	TG:61.7±6.4<br>CG:59.9±7.5	0/60	Isotonic exercise*	2 times	No intervention	12 Weeks	1.VAS<br>2.WOMAC (function)<br>3.Total knee extension 1RM strength<br>4.SF-36 mental health
21	Küçük, et al. 2018	-	TG1:15<br>TG2:15	TG1:52.3±6.9<br>TG2:51.5±5.0	0/30	TG1:Isometric exercise*<br>TG2:Isokinetic exercise*	5 times	-	4 Weeks	1.VAS<br>2.WOMAC (function)
22	Lai, et al. 2021	ChiCTR-IOR-16009234	TG:27<br>CG:27	TG:64.8±4.0<br>CG:63.7± 4.8	6/48	Isometric exercise*	3 times	Health education	8 Weeks	Extensor peak torque at 90°/s
23	Lin, et al. 2009	-	TG:36<br>CG:36	TG: 61.6±7.2<br>CG: 62.2±6.7	22/50	Isotonic exercise*	3 times	No intervention	8 Weeks	1.WOMAC (pain)<br>2.WOMAC (function)<br>3.Extensor peak torque at 60°/s
24	Maharan, et al. 2023	TCTR20230130003	TG:12<br>CG:12	TG:73.8±9.8<br>CG:72.4±8.1	4/20	Isometric exercise*	5 times	No intervention	4 Weeks	1.NRS<br>2.WOMAC (function)<br>3.Isometric contraction strength of the quadriceps

25	Malas, et al. 2013	-	TG1:19<br>TG2:22<br>TG3:20	TG1:59.1±7.2<br>TG2:61.2±8.3<br>TG3:56.2±5.1	10/51	TG1:Isotonic exercise*<br>TG2:Isometric exercise*<br>TG3:Isokinetic exercise*	5 times	-	3 Weeks	1.VAS<br>2.WOMAC (function)<br>3.Extensor peak torque at 60°/s
26	Nahayatbin, et al. 2018	IRCT2014010215936N1	TG:16<br>CG:16	TG:56.1±6.0<br>CG:56.1±6.1	-	Isotonic exercise +Isometric exercise*	3 times	Usual care	4 weeks	1.KOOS (pain)<br>2.KOOS (ADL)<br>3.KOOS (QoL)
27	O'Reilly, et al. 1999	-	TG:108<br>CG:72	TG:61.9±10.0<br>CG:62.2±9.7	61/119	Isotonic exercise +Isometric exercise	7 times	No intervention	24 weeks	1.VAS<br>2.WOMAC (function)<br>3.Isometric contraction strength of the quadriceps<br>4.SF-36 mental health
28	Oh, et al. 2020	-	TG:40<br>CG:20	TG:72.4±6.3<br>CG:71.1±5.4	-	Isotonic exercise +Isometric exercise*	3 times	Health education	20 Weeks	1.WOMAC(pain)<br>2.WOMAC(function)<br>3.Isometric contraction strength of the quadriceps
29	Park, et al. 2021	KCT0006037	TG:27<br>CG:27	TG: 66.9±4.6<br>CG: 68.0±4.2	0/54	Isometric exercise*	3 times	No intervention	8 Weeks	1.KOOS(pain)<br>2.KOOS(ADL)<br>3.KOOS(QoL)<br>4.Extensor peak torque at 60°/s

30	Pazit, et al. 2018	ACTRN12616001382460	TG:9<br>CG:9	TG: 67.8±6.2<br>CG:70.4±7.8	9/9	Isotonic exercise*	2 times	No intervention	8 Weeks	1.WOMAC (pain)<br>2.WOMAC (function)<br>3.3RM Strength Test<br>4.AQoL
31	Rafiq, et al. 2021	IRCT20191221045846N2	TG:25<br>CG:25	TG:53.4±5.2<br>CG:52.8±5.7	23/27	Isotonic exercise	3 times	Usual care	4 weeks	1.WOMAC (pain)<br>2.WOMAC (function)
32	Rogers, et al. 2012	NCT00735098	TG:8<br>CG:8	TG:70.8±6.5<br>CG:71.2±10.9	5/11	Isotonic exercise*	3 times	No intervention	8 Weeks	1.WOMAC (pain)<br>2.WOMAC (function)
33	Salli, et al. 2010	-	TG1:24<br>TG2:23<br>CG:24	TG1:57.1±6.8<br>TG2: 55.7±8.2<br>CG:58.3±6.7	13/58	TG1:Isometric exercise*<br>TG2:Isokinetic exercise*	3 times	Usual care	8 Weeks	1.VAS<br>2.WOMAC (function)<br>3.Extensor peak torque at 60°/s<br>4.SF-36 mental health
34	Samut, et al. 2015	-	TG:15<br>CG:13	TG:62.5±7.7<br>CG:60.9±8.9	0/28	Isokinetic exercise*	3 times	No intervention	6 Weeks	1.VAS<br>2.WOMAC (function)
35	Sayers, et al. 2012	NCT01328340	TG:12<br>CG:11	TG:66.9±4.9<br>CG:68.4±8.1	6/17	Isotonic exercise*	3 times	Usual care	12 Weeks	1.WOMAC (pain)<br>2.WOMAC (function)

36	Schilke, et al. 1996	-	TG:10<br>CG:10	TG:64.5±3.8<br>CG:68.4±8.0	3/17	Isokinetic exercise*	3times	No intervention	8weeks	1.OASI (pain)<br>2.OASI (function)<br>3.Extensor peak torque at 90°/s
37	Simão, et al. 2012	ACTRN126100004 75044	TG:11<br>CG:12	TG:69.0±3.7<br>CG:71.0±5.3	2/21	Isotonic exercise +Isometric exercise*	3 times	No intervention	12 Weeks	1.WOMAC (pain)<br>2.WOMAC (function)
38	Topp, et al. 2002	-	TG1:35<br>TG2:32<br>CG:35	TG1:65.6±1.8<br>TG2: 63.5±1.9<br>CG:60.9±1.8	28/74	TG1:Isotonic exercise*<br>TG2:Isometric exercise*	3 times	No intervention	16 Weeks	1.WOMAC(pain)<br>2.WOMAC(function)
39	Tüzün, et al. 2004	-	TG1:31<br>TG2:31	TG1:60.4±9.1<br>TG2:60.8±8.6	0/62	TG1:Isotonic exercise*<br>TG2:Isokinetic exercise*	5 times	-	2 weeks	1.WOMAC (pain)<br>2.WOMAC (function)<br>3.Extensor peak torque at 60°/s<br>4.SF-36 mental Health
40	Weng, et al. 2009	-	TG:33<br>CG:33	64.0±7.5	-	Isokinetic exercise*	3 times	Usual care	8 Weeks	1.VAS<br>2.Lequesne Index<br>3.Extensor peak torque at 60°/s
41	Wortley, et al. 2013	-	TG:13<br>CG:6	TG:69.5±6.7<br>CG:70.5±5.0	6/13	Isotonic exercise*	2 times	No intervention	10 Weeks	1.WOMAC(pain)<br>2.WOMAC(function)

TG: Treatment group, CG: Control group, *:Conducted under the guidance and supervision of professionals.

Appendix 4: Risk of bias of included studies

Study ID	Selection Bias	Selection Bias	Performance Bias	Detection Bias	Attrition Bias	Reporting Bias	Other bias
Bahşi, et al. 2022	Unclear risk	Unclear risk	High risk	Unclear risk	Low risk	Unclear risk	Unclear risk
Baker, et al. 2001	Low risk	Low risk	High risk	Unclear risk	Low risk	Unclear risk	Unclear risk
Bennell, et al. 2010	Low risk	Low risk	High risk	Low risk	Low risk	Low risk	Low risk
Bruce-Brand, et al. 2012	Low risk	Unclear risk	High risk	Low risk	Unclear risk	Low risk	Low risk
Çakır, et al. 2016	Low risk	Low risk	High risk	Unclear risk	Low risk	Unclear risk	Unclear risk
Chang, et al. 2012	Unclear risk	Unclear risk	High risk	Unclear risk	Low risk	Unclear risk	Unclear risk
Cheing, et al. 2004	Unclear risk	Unclear risk	High risk	Unclear risk	Low risk	Unclear risk	Unclear risk
DeVita, et al. 2018	Low risk	Unclear risk	High risk	Unclear risk	Low risk	Low risk	Low risk
Doss D, et al. 2014	Unclear risk	Unclear risk	High risk	Unclear risk	Low risk	Unclear risk	Unclear risk
Egwu, et al. 2018	Low risk	Low risk	High risk	Unclear risk	Low risk	Unclear risk	Unclear risk
Eyigor, et al. 2004	Unclear risk	Unclear risk	High risk	Low risk	Low risk	Unclear risk	Unclear risk
Foroughi, et al. 2011	Low risk	Low risk	High risk	Low risk	Low risk	Low risk	Low risk
Gür, et al. 2002	Unclear risk	Unclear risk	High risk	Unclear risk	Unclear risk	Unclear risk	Unclear risk
Hsu, et al. 2021	Low risk	Low risk	High risk	Low risk	Low risk	Low risk	Low risk
Huang, et al. 2003	Low risk	Low risk	High risk	Low risk	Low risk	Unclear risk	Unclear risk
Huang, et al. 2005	Low risk	Low risk	High risk	Low risk	Low risk	Unclear risk	Unclear risk
Imoto, et al. 2012	Low risk	Low risk	High risk	Low risk	Low risk	Unclear risk	Unclear risk
Jan, et al. 2008	Low risk	Unclear risk	High risk	Low risk	Low risk	Unclear risk	Unclear risk
Jan, et al. 2009	Low risk	Unclear risk	High risk	Low risk	Low risk	Low risk	Low risk
Jorge, et al. 2015	Low risk	Low risk	High risk	Low risk	Low risk	Unclear risk	Unclear risk
Küçük, et al. 2018	Unclear risk	Unclear risk	High risk	Unclear risk	Low risk	Unclear risk	Unclear risk
Lai, et al. 2021	Low risk	Low risk	High risk	Low risk	Low risk	Low risk	Low risk

Lin, et al. 2009	Low risk	Low risk	High risk	Low risk	Low risk	Unclear risk	Unclear risk
Maharan, et al. 2023	Low risk	Low risk	High risk	Unclear risk	Low risk	Low risk	Low risk
Malas, et al. 2013	Low risk	Unclear risk	High risk	Low risk	Low risk	Unclear risk	Unclear risk
Nahayatbin, et al. 2018	Unclear risk	Unclear risk	High risk	Unclear risk	Low risk	Low risk	Low risk
O'Reilly, et al. 1999	Low risk	Low risk	High risk	Unclear risk	Low risk	Unclear risk	Unclear risk
Oh, et al. 2020	Unclear risk	Unclear risk	High risk	Unclear risk	Unclear risk	Unclear risk	Unclear risk
Park, et al. 2021	Low risk	Unclear risk	High risk	Unclear risk	Low risk	Low risk	Low risk
Pazit, et al. 2018	Low risk	Low risk	High risk	Unclear risk	Low risk	Low risk	Low risk
Rafiq, et al. 2021	Low risk	Unclear risk	High risk	Low risk	Low risk	Low risk	Low risk
Rogers, et al. 2012	Low risk	Unclear risk	High risk	Unclear risk	Unclear risk	Low risk	Low risk
Salli, et al. 2010	Low risk	Low risk	High risk	Low risk	Low risk	Unclear risk	Unclear risk
Samut, et al. 2015	Unclear risk	Unclear risk	High risk	Unclear risk	Low risk	Unclear risk	Unclear risk
Sayers, et al. 2012	Low risk	Unclear risk	High risk	Low risk	Low risk	Low risk	Low risk
Simão, et al. 2012	Low risk	Low risk	High risk	Low risk	Low risk	Low risk	Low risk
Schilke, et al. 1996	Low risk	Unclear risk	High risk	Unclear risk	Low risk	Unclear risk	Unclear risk
Topp, et al. 2002	Unclear risk	Unclear risk	High risk	Unclear risk	Unclear risk	Unclear risk	Unclear risk
Tüzün, et al. 2004	Unclear risk	Unclear risk	High risk	Unclear risk	Unclear risk	Unclear risk	Unclear risk
Weng, et al. 2009	Low risk	Low risk	High risk	Low risk	Low risk	Unclear risk	Unclear risk
Wortley, et al. 2013	Unclear risk	Unclear risk	High risk	Unclear risk	Low risk	Unclear risk	Unclear risk

Appendix 5: Evaluation of inconsistency

Table S5.1: Global consistency

Outcome	χ^2	P value
Pain	13.47	0.26
Function	8.09	0.70
Quality of life	2.33	0.31
Muscle strength	2.55	0.86

Table S5.2: Node-splitting of pain. Inconsistency test between direct and indirect treatment comparisons in mixed treatment comparison.

Comparison	Direct		Indirect		Difference		
	Coef.	Std. Err.	Coef.	Std. Err.	Coef.	Std. Err.	P>|z|
A vs B	0.65	0.09	0.80	0.21	-0.15	0.23	0.52
A vs C	0.67	0.15	0.52	0.18	0.15	0.24	0.53
A vs D	0.81	0.14	0.56	0.16	0.25	0.22	0.25
B vs C	-0.19	0.16	0.08	0.18	-0.26	0.24	0.28
B vs D	-0.04	0.15	0.11	0.16	-0.15	0.22	0.51
C vs D	0.14	0.14	-0.03	0.23	0.16	0.27	0.55

A: Control group, B: Isotonic exercise, C: Isometric exercise, D: Isokinetic exercise.

Table S5.3: Node-splitting of function. Inconsistency test between direct and indirect treatment comparisons in mixed treatment comparison.

Comparison	Direct		Indirect		Difference		
	Coef.	Std. Err.	Coef.	Std. Err.	Coef.	Std. Err.	P>|z|
A vs B	0.62	0.07	0.76	0.18	-0.14	0.20	0.47
A vs C	0.54	0.13	0.60	0.16	-0.06	0.20	0.78
A vs D	0.81	0.12	0.65	0.14	0.16	0.17	0.39
B vs C	-0.23	0.14	0.11	0.15	-0.34	0.20	0.10
B vs D	0.11	0.13	0.11	0.14	0.01	0.19	0.98
C vs D	0.16	0.12	0.22	0.20	-0.06	0.23	0.80

A: Control group, B: Isotonic exercise, C: Isometric exercise, D: Isokinetic exercise.

Table S5.4: Node-splitting of quality of life. Inconsistency test between direct and indirect treatment comparisons in mixed treatment comparison.

Comparison	Direct		Indirect		Difference		
	Coef.	Std. Err.	Coef.	Std. Err.	Coef.	Std. Err.	P>|z|
A vs B	0.35	0.20	0.83	0.45	-0.49	0.49	0.32
A vs C	0.87	0.27	-0.10	0.94	0.98	0.98	0.32
A vs D	0.61	0.41	0.42	0.34	0.19	0.53	0.73
B vs D	-0.07	0.27	0.41	0.41	-0.49	0.49	0.32
C vs D	0.04	0.34	-0.89	0.45	0.92	0.57	0.11

A: Control group, B: Isotonic exercise, C: Isometric exercise, D: Isokinetic exercise.

Table S5.5: Node-splitting of muscle strength. Inconsistency test between direct and indirect treatment comparisons in mixed treatment comparison.

Comparison	Direct		Indirect		Difference		
	Coef.	Std. Err.	Coef.	Std. Err.	Coef.	Std. Err.	P>|z|
A vs B	0.53	0.10	0.34	0.19	0.19	0.21	0.38
A vs C	0.34	0.14	0.45	0.19	-0.11	0.23	0.63
A vs D	0.53	0.18	0.59	0.15	-0.06	0.23	0.81
B vs C	0.09	0.22	-0.19	0.15	0.28	0.27	0.29
B vs D	0.11	0.15	0.02	0.18	0.09	0.23	0.69
C vs D	0.15	0.14	0.26	0.22	-0.11	0.26	0.68

A: Control group, B: Isotonic exercise, C: Isometric exercise, D: Isokinetic exercise.

Appendix 6: League table and SUCRA

Figure S6.1.1: The league table of different lower limb strengthening exercises for reducing pain

**Mixed exercise**				
-0.23 (-0.54,0.08)	**Isokinetic exercise**			
-0.13 (-0.46,0.19)	0.09 (-0.14,0.32)	**Isometric exercise**		
-0.20 (-0.48,0.08)	0.03 (-0.19,0.24)	-0.07 (-0.30,0.17)	**Isotonic exercise**	
0.48 (0.24,0.71)	0.70 (0.50,0.91)	0.61 (0.38,0.84)	0.68 (0.52,0.84)	**Control group**

Notes: Mixed exercise, Mixed strengthening exercise (The combination of different types of lower limb muscle contraction methods in strengthening exercises).

Figure S6.1.2: The surface under the cumulative ranking curve plots of different lower limb strengthening exercises for reducing pain

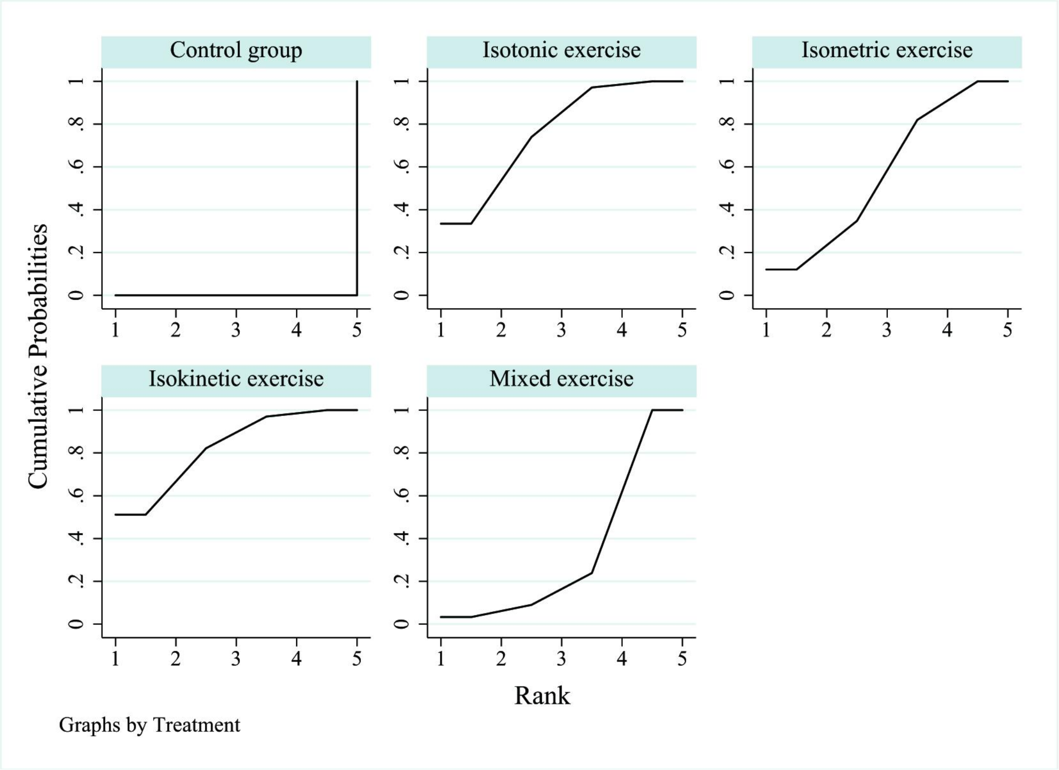

Notes: Mixed exercise, Mixed strengthening exercise (The combination of different types of lower limb muscle contraction methods in strengthening exercises).

Table S6.1: SUCRA of the effects of different lower limb strengthening exercises for reducing pain

Treatment	SUCRA(%)	PrBest	MeanRank
Control group	0.0	0.0	5.0
Isotonic exercise	76.2	32.9	2.0
Isometric exercise	57.2	12.9	2.7
Isokinetic exercise	82.6	50.7	1.7
Mixed strengthening exercise	34.0	3.4	3.6

Figure S6.2.1: The league table of different lower limb strengthening exercises for improving function

**Mixed exercise**				
-0.34 (-0.60,-0.08)	**Isokinetic exercise**			
-0.16 (-0.43,0.11)	0.18 (-0.02,0.38)	**Isometric exercise**		
-0.23 (-0.46,-0.01)	0.11 (-0.08,0.29)	-0.07 (-0.27,0.13)	**Isotonic exercise**	
0.41 (0.22,0.59)	0.75 (0.57,0.92)	0.57 (0.37,0.76)	0.64 (0.51,0.77)	**Control group**

Notes: Mixed exercise, Mixed strengthening exercise (The combination of different types of lower limb muscle contraction methods in strengthening exercises).

Figure S6.2.2: The surface under the cumulative ranking curve plots of different lower limb strengthening exercises for improving function

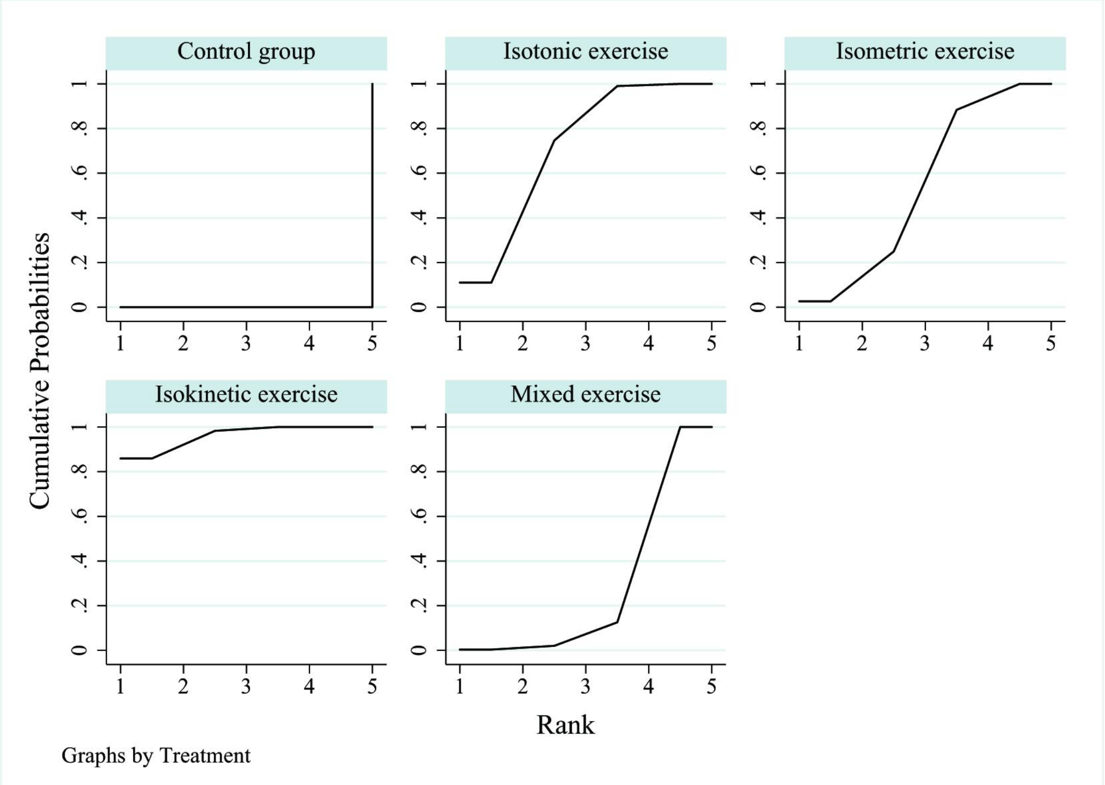

Notes: Mixed exercise, Mixed strengthening exercise (The combination of different types of lower limb muscle contraction methods in strengthening exercises).

Table S6.2: SUCRA of the effects of different lower limb strengthening exercises for improving function

Treatment	SUCRA(%)	PrBest	MeanRank
Control group	0.0	0.0	5.0
Isotonic exercise	71.2	11.6	2.1
Isometric exercise	54.0	2.8	2.9
Isokinetic exercise	96.1	85.4	1.2
Mixed strengthening exercise	28.7	0.2	3.8

Figure S6.3.1: The league table of different lower limb strengthening exercises for improving quality of life

**Mixed exercise**				
-0.13 (-0.79,0.52)	**Isokinetic exercise**			
-0.43 (-1.11,0.25)	-0.30 (-0.90,0.30)	**Isometric exercise**		
-0.06 (-0.63,0.51)	0.07 (-0.37,0.51)	0.37 (-0.21,0.95)	**Isotonic exercise**	
0.36 (-0.09,0.81)	0.50 (0.02,0.98)	0.80 (0.28,1.31)	0.43 (0.07,0.78)	**Control group**

Notes: Mixed exercise, Mixed strengthening exercise (The combination of different types of lower limb muscle contraction methods in strengthening exercises).

Figure S6.3.2: The surface under the cumulative ranking curve plots of different lower limb strengthening exercises for improving quality of life

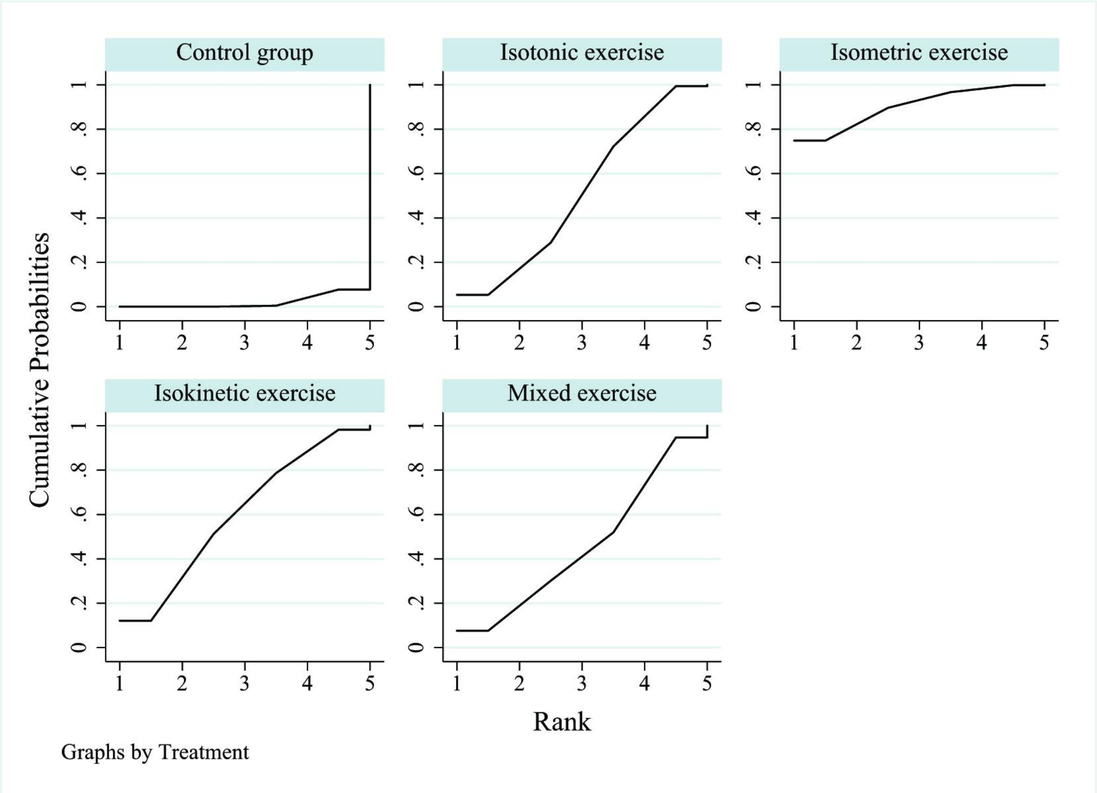

Notes: Mixed exercise, Mixed strengthening exercise (The combination of different types of lower limb muscle contraction methods in strengthening exercises).

Table S6.3: SUCRA of the effects of different lower limb strengthening exercises for improving quality of life

Treatment	SUCRA(%)	PrBest	MeanRank
Control group	2.4	0.0	4.9
Isotonic exercise	50.9	5.4	2.9
Isometric exercise	90.5	75.0	1.4
Isokinetic exercise	60.1	12.4	2.6
Mixed strengthening exercise	46.1	7.3	3.2

Figure S6.4.1: The league table of different lower limb strengthening exercises for enhancing muscle strength

**Mixed exercise**				
-0.22 (-0.54,0.09)	**Isokinetic exercise**			
-0.04 (-0.36,0.27)	0.18 (-0.05,0.41)	**Isometric exercise**		
-0.15 (-0.44,0.14)	0.08 (-0.15,0.30)	-0.11 (-0.35,0.14)	**Isotonic exercise**	
0.34 (0.11,0.56)	0.56 (0.34,0.78)	0.38 (0.16,0.60)	0.49 (0.31,0.66)	**Control group**

Notes: Mixed exercise, Mixed strengthening exercise (The combination of different types of lower limb muscle contraction methods in strengthening exercises).

Figure S6.4.2: The surface under the cumulative curve plots of different lower limb strengthening exercises for enhancing muscle strength

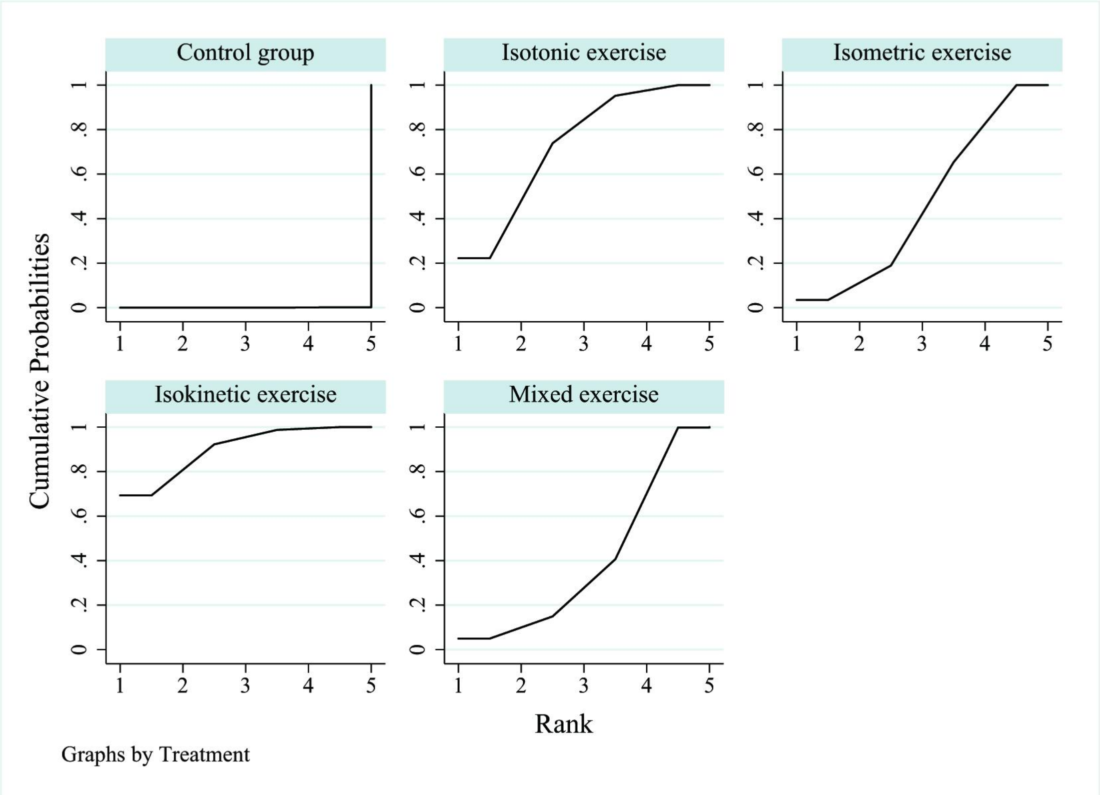

Notes: Mixed exercise, Mixed strengthening exercise (The combination of different types of lower limb muscle contraction methods in strengthening exercises).

Table S6.4: SUCRA of the effects of different lower limb strengthening exercises for enhancing muscle strength

Treatment	SUCRA(%)	PrBest	MeanRank
Control group	0.1	0.0	5.0
Isotonic exercise	72.8	23.4	2.1
Isometric exercise	46.9	3.4	3.1
Isokinetic exercise	90.1	67.8	1.4
Mixed strengthening exercise	40.1	5.3	3.4

Appendix 7: Funnel plots

Figure S7.1: Funnel plot of different lower limb strengthening exercises for reducing pain

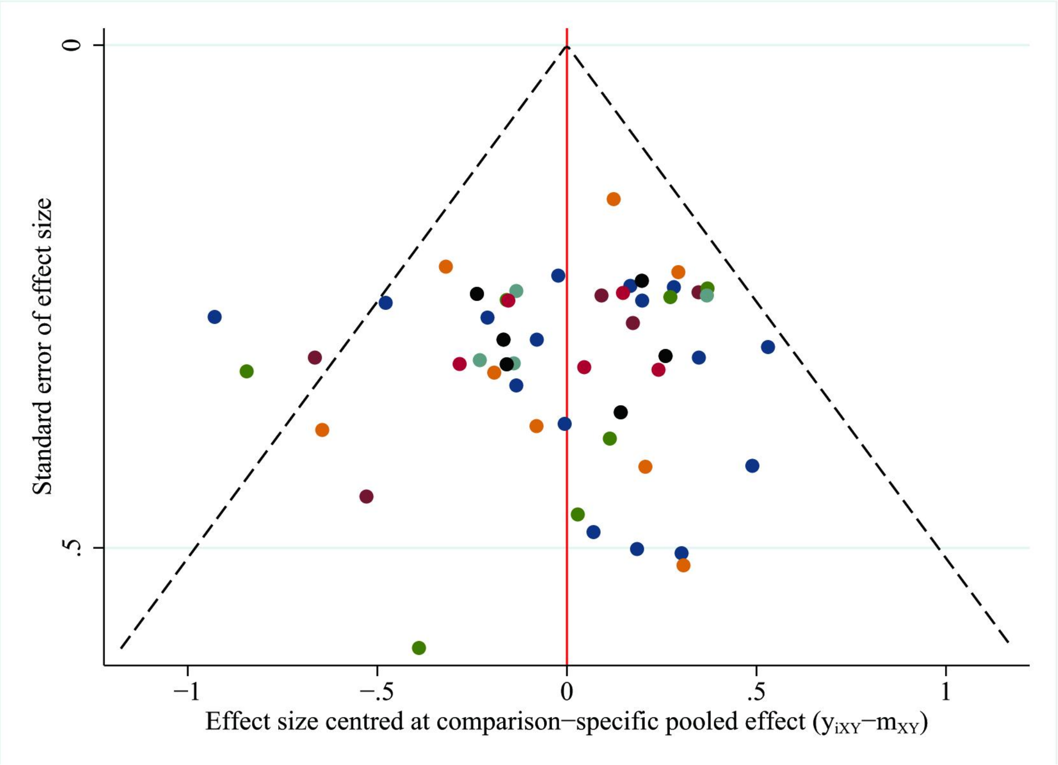

Figure S7.2: Funnel plot of different lower limb strengthening exercises for improving function

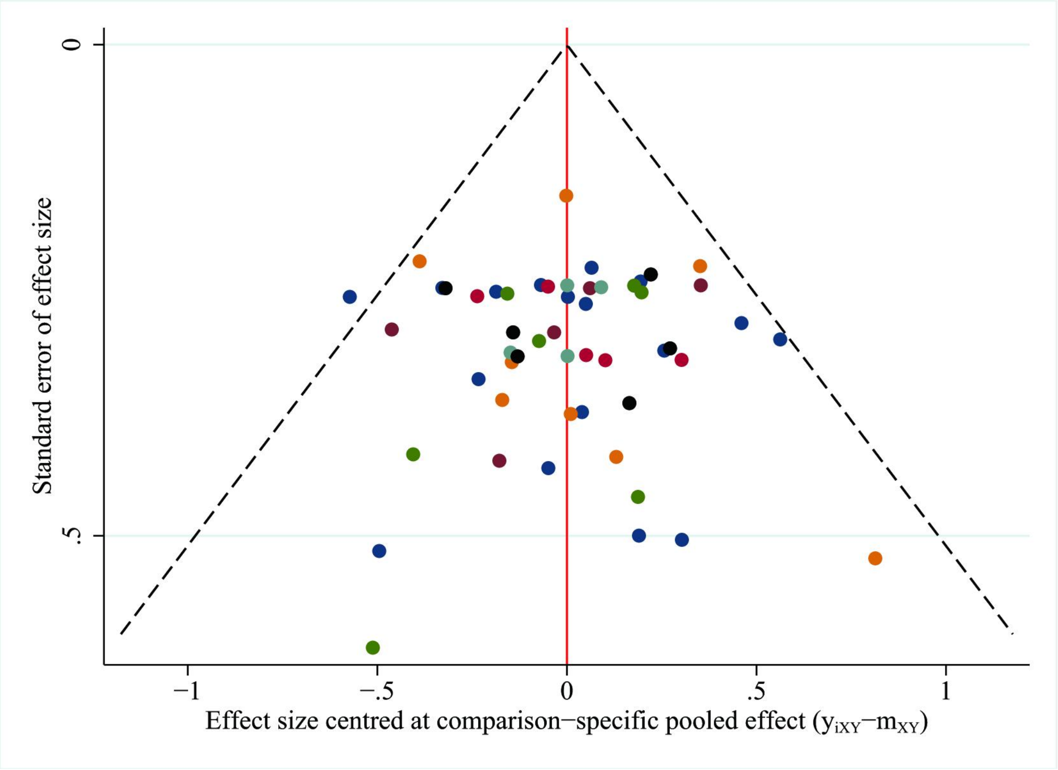

Figure S7.3: Funnel plot of different lower limb strengthening exercises for improving quality of life

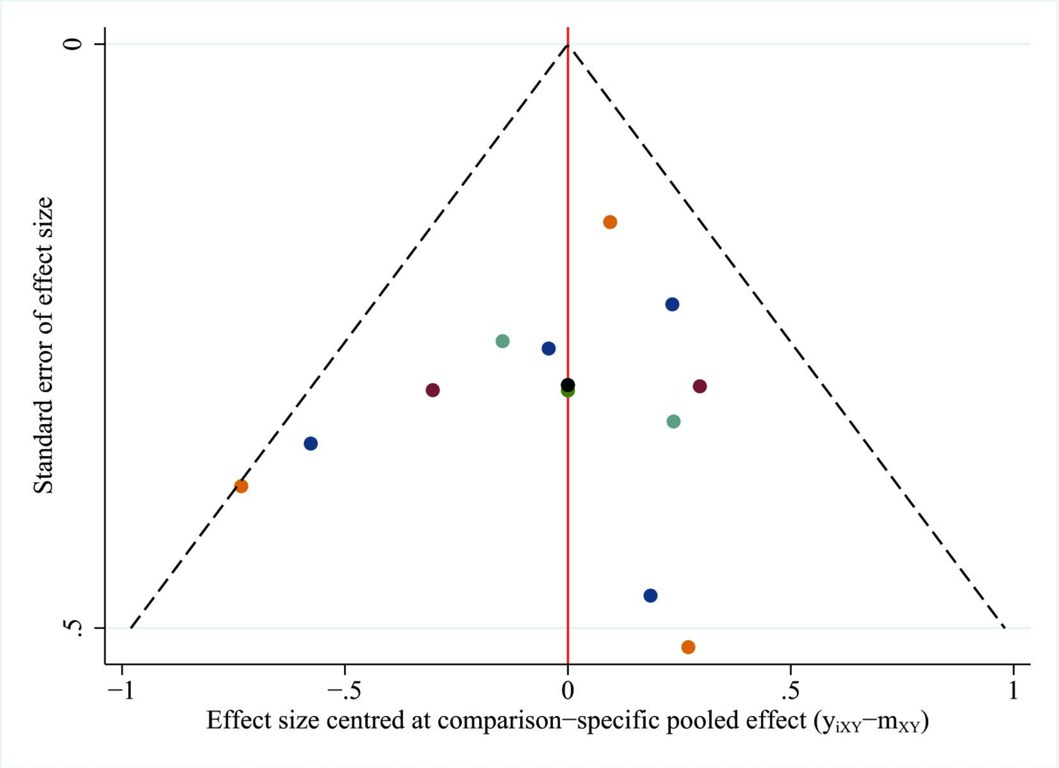

Figure S7.4: Funnel plot of different lower limb strengthening exercises for enhancing muscle strength

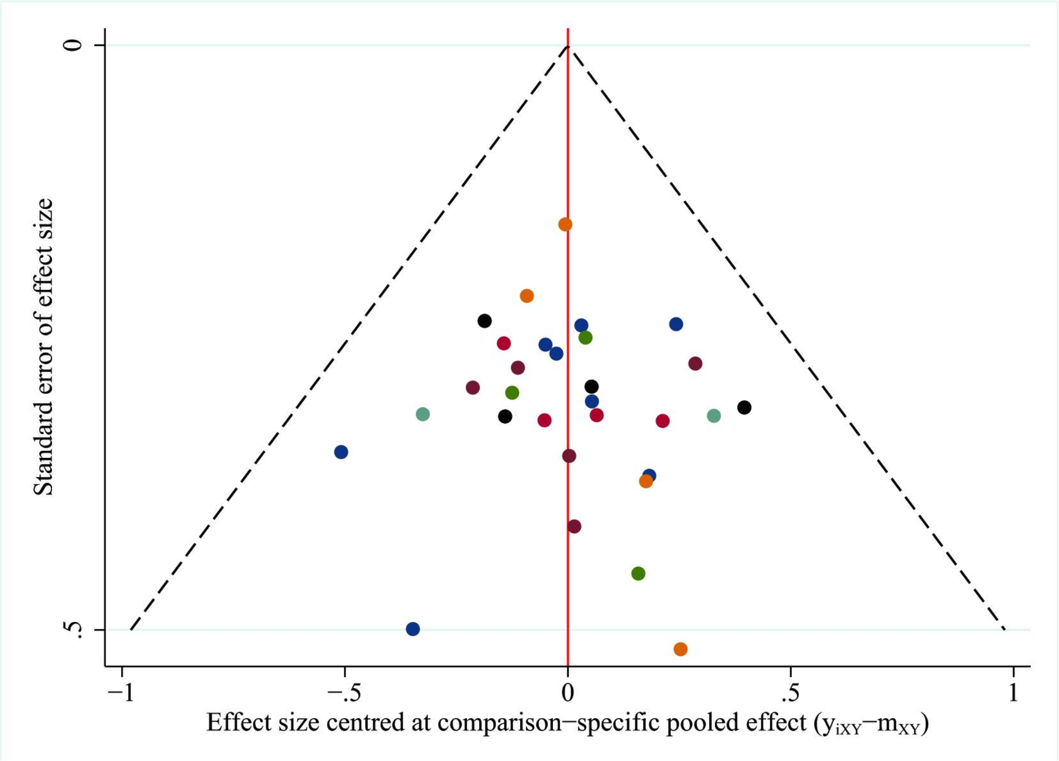

Appendix 8: Results of paired comparison meta-analysis

Table S8.1: Results of paired comparison meta-analysis on pain

Treatment type	Studies	n	SMD (95%CI)	P-value	I^2 (%)	Egger's P
B VS A	16	767	0.67 (0.46 to 0.87)	<0.001	43.4	0.52
C VS A	5	259	0.72 (0.36 to 1.09)	<0.001	50.1	0.13
D VS A	7	311	0.87 (0.52 to 1.23)	<0.001	51.4	0.34
E VS A	8	492	0.48 (0.26 to 0.69)	<0.001	18.8	0.55

A: Control group, B: Isotonic exercise, C: Isometric exercise, D: Isokinetic exercise, E:Mixed strengthening exercise.

Table S8.2: Results of paired comparison meta-analysis on function

Treatment type	Studies	n	SMD (95%CI)	P-value	I^2 (%)	Egger's P
B VS A	17	838	0.63 (0.49 to 0.78)	<0.001	10.0	0.78
C VS A	5	259	0.55 (0.27 to 0.84)	<0.001	22.5	0.35
D VS A	7	311	0.84 (0.60 to 1.07)	<0.001	0.0	0.22
E VS A	8	492	0.41 (0.19 to 0.62)	<0.001	20.2	0.56

A: Control group, B: Isotonic exercise, C: Isometric exercise, D: Isokinetic exercise, E: Mixed strengthening exercise.

Table S8.3: Results of paired comparison meta-analysis on quality of life

Treatment type	Studies	n	SMD (95%CI)	P-value	I^2 (%)	Egger's P
B VS A	4	197	0.35 (-0.01 to 0.70)	0.054	30.4	0.61
C VS A	2	102	0.90 (0.34 to 1.47)	0.002	47.1	-
D VS A	1	47	0.60 (0.01 to 1.18)	0.046	-	-
E VS A	3	228	0.40 (-0.17 to 0.97)	0.169	57.2	0.70

A: Control group, B: Isotonic exercise, C: Isometric exercise, D: Isokinetic exercise, E: Mixed strengthening exercise.

Table S8.4: Results of paired comparison meta-analysis on muscle strength

Treatment type	Studies	n	SMD (95%CI)	P-value	I^2 (%)	Egger's P
B VS A	8	398	0.54 (0.34 to 0.74)	<0.001	0.0	0.16
C VS A	5	213	0.35 (0.07 to 0.62)	0.013	0.0	0.84
D VS A	3	113	0.56 (0.21 to 0.90)	0.002	0.0	0.83
E VS A	4	317	0.34 (0.11 to 0.57)	0.003	0.0	0.25

A: Control group, B: Isotonic exercise, C: Isometric exercise, D: Isokinetic exercise, E: Mixed strengthening exercise.

Appendix 9: Results under different intervention frequencies for pain

S9.1: Network evidence map

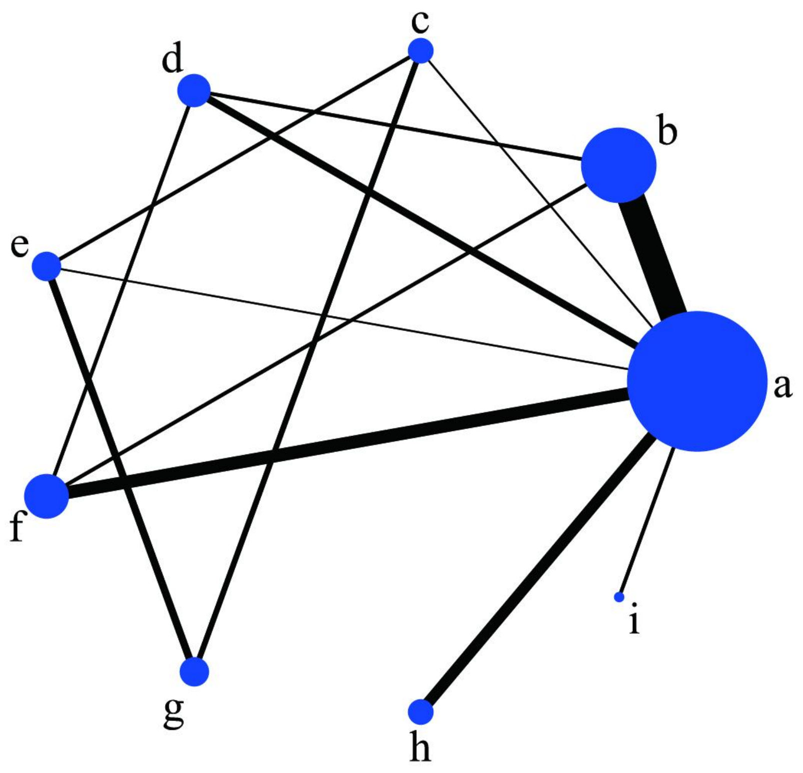

a: Control group, b: Isotonic exercise (low-frequency), c: Isotonic exercise (high-frequency), d: Isometric exercise (low-frequency), e: Isometric exercise (high-frequency), f: Isokinetic exercise (low-frequency), g: Isokinetic exercise (high-frequency), h: Mixed strengthening exercise (low-frequency), i: Mixed strengthening exercise (high-frequency).

S9.2: Global consistency

$\chi^2 = 19.7$ P = 0.055

Table S9.2: Node-splitting of pain. Inconsistency test between direct and indirect treatment comparisons in mixed treatment comparison.

Comparison	Direct		Indirect		Difference		
	Coef.	Std. Err.	Coef.	Std. Err.	Coef.	Std. Err.	P>|z|
a vs b	0.58	0.08	1.01	0.29	-0.43	0.31	0.16
a vs c	1.59	0.29	1.14	0.50	0.45	0.58	0.44
a vs d	0.60	0.14	-0.10	0.30	0.71	0.33	0.03
a vs e	1.19	0.46	1.63	0.35	-0.44	0.58	0.44
a vs f	0.79	0.12	0.13	0.26	0.65	0.29	0.02
b vs d	-0.31	0.19	0.06	0.20	-0.37	0.27	0.18
b vs f	-0.22	0.21	0.22	0.16	-0.44	0.26	0.09
c vs e	-0.01	0.24	0.00	0.29	-0.01	0.37	0.98
c vs g	0.09	0.18	-0.31	0.46	0.40	0.50	0.42
d vs f	0.32	0.20	0.03	0.23	0.29	0.30	0.33
e vs g	0.02	0.16	0.37	0.53	-0.35	0.55	0.53

a: Control group, b: Isotonic exercise (low-frequency), c: Isotonic exercise (high-frequency), d: Isometric exercise (low-frequency), e: Isometric exercise (high-frequency), f: Isokinetic exercise (low-frequency), g: Isokinetic exercise (high-frequency).

S9.3: The league table

**Mixed exercise (high-frequency)**								
0.05<br>(-0.35,0.46)	**Mixed exercise (low-frequency)**							
-1.03<br>(-1.65,-0.41)	-1.08<br>(-1.70,-0.46)	**Isokinetic exercise (high-frequency)**						
-0.18<br>(-0.54,0.18)	-0.24<br>(-0.60,0.12)	0.85<br>(0.25,1.44)	**Isokinetic exercise (low-frequency)**					
-0.98<br>(-1.60,-0.37)	-1.04<br>(-1.65,-0.42)	0.05<br>(-0.25,0.34)	-0.80<br>(-1.39,-0.21)	**Isometric exercise (high-frequency)**				
0.01<br>(-0.37,0.39)	-0.04<br>(-0.42,0.34)	1.04<br>(0.44,1.65)	0.20<br>(-0.10,0.49)	0.99<br>(0.40,1.59)	**Isometric exercise (low-frequency)**			
-0.99<br>(-1.56,-0.42)	-1.04<br>(-1.61,-0.48)	0.04<br>(-0.29,0.37)	-0.81<br>(-1.34,-0.27)	-0.01<br>(-0.36,0.35)	-1.00<br>(-1.55,-0.45)	**Isotonic exercise (high-frequency)**		
-0.12<br>(-0.45,0.20)	-0.18<br>(-0.50,0.15)	0.91<br>(0.33,1.48)	0.06<br>(-0.19,0.31)	0.86<br>(0.30,1.42)	-0.14<br>(-0.40,0.13)	0.86<br>(0.35,1.38)	**Isotonic exercise (low-frequency)**	
0.49<br>(0.20,0.78)	0.44<br>(0.15,0.72)	1.52<br>(0.97,2.07)	0.67<br>(0.45,0.89)	1.47<br>(0.93,2.01)	0.48<br>(0.23,0.73)	1.48<br>(0.99,1.97)	0.61<br>(0.46,0.77)	**Control group**

Notes: Mixed exercise, Mixed strengthening exercise (The combination of different types of lower limb muscle contraction methods in strengthening exercises).

S9.4: The surface under the cumulative curve plots of different intervention frequencies for reducing pain

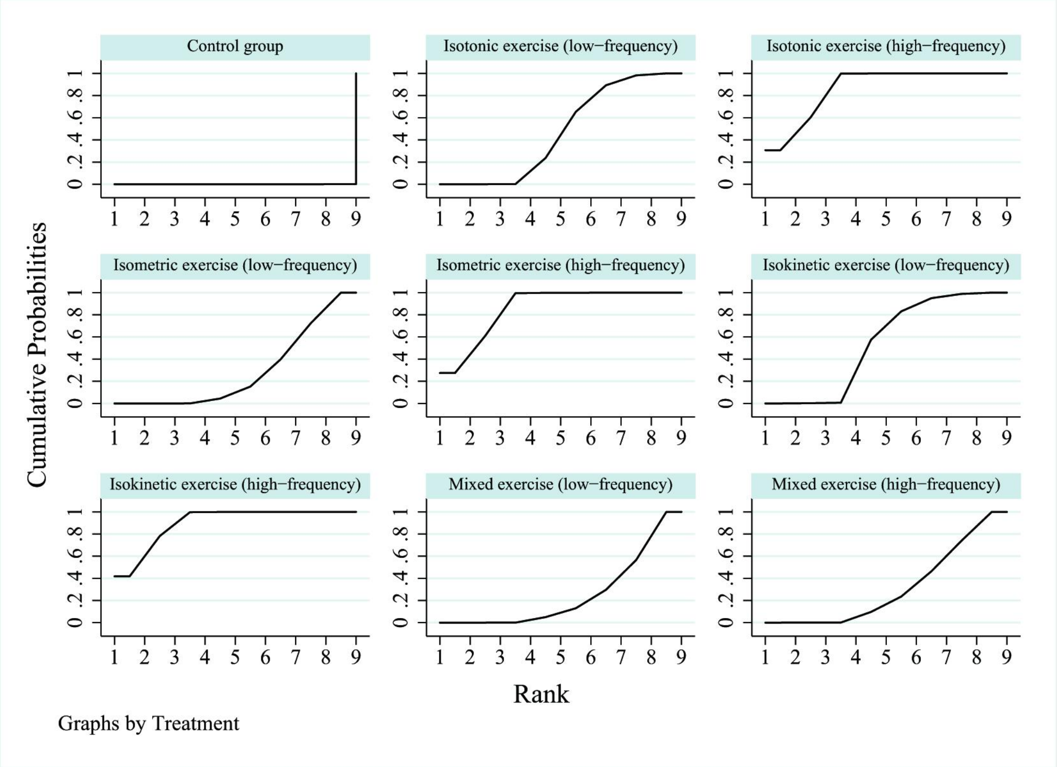

Notes: Mixed exercise, Mixed strengthening exercise (The combination of different types of lower limb muscle contraction methods in strengthening exercises).

S9.5: SUCRA of the effects of different intervention frequencies for reducing pain

Treatment	SUCRA(%)	PrBest	MeanRank
Control group	0.0	0.0	9.0
Isotonic exercise (low-frequency)	47.1	0.0	5.2
Isotonic exercise (high-frequency)	86.4	30.7	2.1
Isometric exercise (low-frequency)	29.0	0.0	6.7
Isometric exercise (high-frequency)	86.0	27.5	2.1
Isokinetic exercise (low-frequency)	54.4	0.0	4.6
Isokinetic exercise (high-frequency)	89.9	41.8	1.8
Mixed strengthening exercise (low-frequency)	25.5	0.0	7.0
Mixed strengthening exercise (high-frequency)	31.7	0.0	6.5

Appendix 10: Specific details of the exercise prescription included in the study

Study ID	Types	Intensity	Frequency	Duration	Supervision
Bahşi, et al. 2022	Weightlifting exercises with 1.5 kg weight (Isotonic exercise)	90 repetitions with 1.5 kg weight	5 times/week	3 weeks	Yes
	Static muscle contraction (Isometric exercises)	Maximal effort for 10 seconds	5 times/week	3 weeks	Yes
	Concentric-concentric flexion and extension (Isokinetic exercise)	10 repetitions at 60°/s and 180°/s velocities	5 times/week	3 weeks	Yes
Baker, et al. 2001	Squats, Step-ups, Isotonic exercises (knee extensions, knee flexions, hip extensions, etc.) using ankle weights (Isotonic exercise)	Start at light intensity (Borg scale 3-5) and progress to a higher intensity (up to Borg scale 8); 2 sets of 12 repetitions for each exercise	3 times/week	16 weeks	Yes
Bennell, et al. 2010	Hip abductor and adductor muscle strengthening exercises (e.g., abduction, adduction, isometric holds) (Isotonic exercise + Isometric exercise)	3 sets of 10 repetitions, resistance adjusted to 10 RM or moderate resistance	5 times/week	12 weeks	Yes
Bruce-Brand, et al. 2012	Knee presses, extended leg raises, wall squats, hamstring curls, using Thera-Bands (Isotonic exercise + Isometric exercise)	3 sets of 10 repetitions per exercise (approx. 30 mins per session)	3 times/week	6 weeks	Yes

Çakır, et al. 2016	Isometric strengthening exercises for quadriceps and adductor muscles<br>(Isometric exercise)	3 sets of 10 repetitions per muscle group, 10 seconds rest between sets	5 times/week	2 weeks	Yes
	Isokinetic concentric exercises for knee flexors and extensors<br>(Isokinetic exercise)	1 set of 10 repetitions at 120°/s and 90°/s, with 20 seconds rest between sets	5 times/week	2 weeks	Yes
Chang, et al. 2012	Elastic band strength training: The leg gradually bends from a straight position, then fully extends and holds, and finally bends again.<br>(Isotonic exercise + Isometric exercise)	10 repetitions per set, 3 sets per session, with resistance bands of increasing intensity	3 times/week	8 weeks	Yes
Cheing, et al, 2004	Isometric exercise training for knee extensors and flexors<br>(Isometric exercise)	6 maximal 5-second isometric contractions at 30°, 60°, 90°	5 times/week	4 weeks	Yes
DeVita, et al, 2018	Leg extensions, leg press, forward lunge exercises<br>(Isotonic exercise)	3 sets of 10 repetitions per exercise, progressed from 60% to 85% 3RM	3 times/week	12 weeks	Yes
Doss D,et al.2014	Knee extensions with eccentric quadriceps contraction<br>(Isotonic exercise)	5 sets of 10 repetitions, 50% of 1RM, incremented by 5% each week	5 times/week	4 weeks	Yes
Egwu, et al. 2018	1. Quadriceps Setting: The participant lies in a supine position, contracts the quadriceps, keeping the knee fully extended, holding for 10 seconds.<br>2. Straight Leg Raising (SLR): The participant lifts the straight leg up to about 45° while maintaining the knee extended, holding briefly, and lowering.<br>3. Mini-Squats: The participant performs partial squats (30–60° knee flexion), keeping the trunk upright.	10 repetitions per exercise, progressively increased resistance	3 times/week	6 weeks	Yes

	(Isotonic exercise + Isometric exercise)				
Eyigor, et al. 2004	Progressive resistive knee extensions using De Lormé method (Isotonic exercise)	3 sets of 10 repetitions: 1st set at 50% of 10RM, 2nd set at 75%, 3rd set at full 10RM	5 times/week	6 weeks	Yes
	Isokinetic knee extensions and flexions at 60°, 90°, 120°, and 180°/s angular velocities (Isokinetic exercise)	3 sets of 6 repetitions at each angular velocity (60°, 90°, 120°, 180°/s)	3 times/week	6 weeks	Yes
Foroughi, et al. 2011	Unilateral knee extensions, standing hip abduction and adduction, bilateral knee flexion, leg press, and plantar-flexion (Isotonic exercise)	3 sets of 8 repetitions at 80% of 1RM, increased by 3% per session or adjusted fortnightly by 1RM tests	3 times/week	24 weeks	Yes
Gür, et al. 2002	Concentric and eccentric knee extensions and flexions with isokinetic contractions (Isokinetic exercise)	6 concentric and 6 eccentric contractions of knee extensor and flexor muscles at angular velocities of 30°/s to 180°/s (30° intervals)	3 times/week	8 weeks	Yes
Hsu, et al. 2021	Seated, open-chain elastic band exercises: hip extension/flexion, abduction/adduction, external/internal rotation, knee extension/flexion, ankle plantarflexion/dorsiflexion (Isotonic exercise)	10 repetitions/set, 5 sets/day, gradually increasing resistance with thicker bands	3 times/week	12 weeks	Yes
Huang, et al. 2003	Isotonic knee extensions and flexions with variable speed (Isotonic exercise)	5 repetitions of maximal effort, variable speed	3 times/week	8 weeks	Yes
	Isometric knee exercises with static contraction (Isometric exercise)	5 seconds hold in different angles of motion for each set	3 times/week	8 weeks	Yes
	Isokinetic knee extensions and flexions with constant speed (Isokinetic exercise)	5 sets of 5 repetitions at 30°/s and 120°/s angular velocity	3 times/week	8 weeks	Yes

Huang, et al. 2005	Isokinetic knee flexion and extension exercises (Isokinetic exercise)	5 sets per session, with 5 repetitions of concentric and eccentric contractions at 30°/s and 120°/s angular velocity	3 times/week	8 weeks	Yes
Imoto, et al. 2012	Quadriceps strengthening exercises: seated knee extensions (Isotonic exercise)	3 sets of 15 repetitions with 50-60% of 10 RM, progressive load increase as tolerated	2 times/week	8 weeks	Yes
Jan, et al. 2008	Leg press using the EN-Dynamic Track leg press machine (Isotonic exercise)	60% of 1RM, 3 sets of 8 repetitions	3 times/week	8 weeks	Yes
Jan, et al. 2009	Knee extension-flexion exercises performed in a sitting position with foot fixed on a pedal for resistance (Isotonic exercise)	4 sets of 6 repetitions at 50% of 1RM, progressively increasing by 5% every 2 weeks	3 times/week	8 weeks	Yes
Jorge, et al. 2015	Strengthening exercises for knee extensors, flexors, hip abductors, and adductors using machines with free weights (Isotonic exercise)	2 sets of 8 repetitions using 50% and 70% of 1RM, progressively increasing load every two weeks	2 times/week	12 weeks	Yes
Küçük, et al. 2018	Isometric quadriceps contraction exercises (Isometric exercise)	10 repetitions of straight leg raises and quadriceps isometric contractions held for 10 seconds	5 times/week	4 weeks	Yes
	Isokinetic knee extension and flexion exercises (Isokinetic exercise)	10 repetitions of concentric-concentric flexion and extension contractions at velocities of 60°, 90°, 120°, 150°, and 180°/s	5 times/week	4 weeks	Yes
Lai, et al. 2021	Static squats performed on flat ground (Isometric exercise)	3 sets of 6 repetitions	3 times/week	8 weeks	Yes

Lin, et al. 2009	Seated knee extensions using an En-Dynamic dynamometer with concentric and eccentric contractions (Isotonic exercise)	4 sets of 6 repetitions at 50% of 1RM, increased by 5% of 1RM every 2 weeks	3 times/week	8 weeks	Yes
Maharan, et al. 2023	Home-based isometric quadriceps exercise: Maximum active contraction of the quadriceps while seated, with a plastic spring-like device beneath the affected knee (Isometric exercise)	10 repetitions per set, 3 sets per day, holding each contraction for 10 seconds	5 times/week	4 weeks	Yes
Malas, et al. 2013	Knee extensions with 1.5 kg weight at variable speed (Isotonic exercise)	90 repetitions per session	5 times/week	3 weeks	Yes
	Isometric knee extensions held at maximal effort for 10 seconds (Isometric exercise)	90 repetitions per session	5 times/week	3 weeks	Yes
	Isokinetic knee flexion/extension at 60°, 120°, and 240°/s (Isokinetic exercise)	90 repetitions per session	5 times/week	3 weeks	Yes
Nahayatbin, et al. 2018	1.Standing terminal extension: Holding the knee in full extension for 10 seconds.<br>2.Mini squats: Performing squats with 15° knee flexion.<br>3.Front and side step-ups: Stepping up and down from a platform.<br>4.Lunges: Forward lunges with 10-second holds.<br>(Isotonic exercise + Isometric exercise)	20 minutes per session with 10-second holds and rests for each movement	3 times/week	4 weeks	Yes
O'Reilly, et al. 1999	Isometric contraction of quadriceps (full extension, pushing against a towel), isotonic quadriceps contraction, hamstring contraction, dynamic stepping exercises (Isotonic exercise + Isometric exercise)	Up to 20 repetitions per leg, with five-second holds for quadriceps and hamstring contractions, progression in intensity based on individual capacity	Daily	24 weeks	Unclear

Oh, et al. 2020	Resistance training using a loop band (TheraBand), performed in a sitting, standing, and lying position, with movements for warming up, main exercises, and cool down. (Isotonic exercise + Isometric exercise)	Low-resistance yellow loop band, gradually increasing repetitions every 4 weeks. Sessions were 2-3 times per week.	3 times/week	20 weeks	Yes
Park, et al. 2021	Isometric exercise (e.g., crunches, bridges, leg raises, planks, lunges, squats) (Isometric exercise)	6-second contraction followed by 4-second rest, with electrical stimulation intensity at 60%, 70%, and 80% of 1 maximal tolerance (1MT) over 8 weeks.	3 times/week	8 weeks	Yes
Pazit, et al. 2018	High-speed resistance training (HSRT) including exercises such as leg press, sit to stand, squat, step-ups, calf raises, lunges, stair climbing (Isotonic exercise)	Phased progression over 8 weeks: 20-40% of 1RM for 8-12 reps in weeks 1-2; 40-60% of 1RM for 5-8 reps in weeks 3-5; 60-80% of 1RM for 2-5 reps in weeks 6-8.	2 times/week	8 weeks	Yes
Rafiq, et al. 2021	Strengthening exercises of the lower limb rehabilitation protocol (LLRP) in non-weight-bearing positions, including resistance band exercises for hip, quadriceps, hamstrings, and ankle. (Isotonic exercise)	2 sets of 7 reps (1st-2nd weeks), progressing to 2 sets of 10 reps (3rd-4th weeks) with a resistance band or ankle weights depending on the muscle group.	3 times/week	4 weeks	Unclear

Rogers, et al. 2012	Resistance training using Thera-Band® non-latex elastic resistance bands, focusing on seated and standing open-chain exercises such as ankle extension, ankle flexion, knee extension, knee flexion, hip abduction, hip adduction, hip internal and external rotation, and leg press (hip and knee extension).<br>(Isotonic exercise)	Each exercise was performed for 15 repetitions per leg. If participants couldn't complete 15 reps, they were advised to perform as many as possible, progressing towards 15. The intensity was increased by adding more stretch to the resistance band or moving to a stronger resistance band.	3 times/week	8 weeks	Yes
Salli, et al. 2010	Isometric exercises for knee muscles.<br>(Isometric exercise)	Performed using an isokinetic dynamometer at submaximal effort levels.	3 times/week	8 weeks	Yes
	Concentric-Eccentric isokinetic exercises for knee muscles<br>(Isokinetic exercise)	10 repetitions at angular velocities (60, 90, 120, 150, and 180°/s) with 70% of maximal voluntary contraction.	3 times/week	8 weeks	Yes
Samut, et al. 2015	Isokinetic exercises with flexion and extension movements at different angular velocities (60°, 90°, 120°, and 180°/s).<br>(Isokinetic exercise)	One set initially, progressing to 6 sets per session with 20 seconds rest between sets.	3 times/week	6 weeks	Yes
Sayers, et al. 2012	High-speed leg press and knee extension exercises performed explosively during the concentric phase and controlled during the eccentric phase.<br>(Isotonic exercise)	3 sets of 12–14 repetitions at 40% of 1RM	3 times/week	12 weeks	Yes
Schilke, et al. 1996	Isokinetic strength training using a Cybex II dynamometer for knee extension and flexion.<br>(Isokinetic exercise)	6 sets of 5 maximal contractions at 90° per second for each leg.	3 times/week	8 weeks	Yes

Simão, et al. 2012	Squats with knee flexion from 10° to 60° without vibration. Isometric contraction during the 3-second hold at 60° knee flexion.<br>(Isotonic exercise + Isometric exercise)	Progressed to 8 sets, with rest periods of 20-40 seconds.	3 times/week	12 weeks	Yes
Topp, et al. 2002	Dynamic resistance exercises using Thera-Band elastic bands: Full range of motion exercises for the same muscle groups, including concentric and eccentric phases<br>(Isotonic exercise)	1 set of 8 repetitions initially, progressing to 3 sets of 12 repetitions with increasing band resistance.	3 times/week	16 weeks	Yes
	Isometric resistance exercises: Static holds performed at specific joint angles (knee flexion at 10°) for various muscle groups including knee extensors, knee flexors, ankle dorsiflexors, and plantar flexors.<br>(Isometric exercise)	1 set of 8 repetitions with muscle tension held for 3-5 seconds. Progressed to 3 sets of 12 repetitions, with maximal tension in the latter	3 times/week	16 weeks	Yes
Tüzün, et al. 2004	Isotonic exercises for quadriceps strengthening based on the Delorme technique, involving progressive resistance training.<br>(Isotonic exercise)	Exercises started with light resistance and progressed to maximal levels.	5 times/week	2 weeks	Yes
	Isokinetic exercises for knee flexion and extension using a Cybex dynamometer. Movements performed at different angular velocities (60, 90, 120, and 180°/s).<br>(Isokinetic exercise)	10 repetitions at each angular velocity with a 60-second rest between sets.	5 times/week	2 weeks	Yes
Weng, et al. 2009	Isokinetic knee flexion and extension exercises.<br>(Isokinetic exercise)	5 repetitions at angular velocities of 30°/s and 120°/s for both concentric and eccentric contractions, starting at 60% of peak torque. Progressively increased to 6 sets.	3 times/week	8 weeks	Yes

Wortley, et al. 2013	Open-kinetic chain resistance training: exercises included seated leg extensions, standing hamstring curls, straight leg raises, standing hip abductions, standing hip adductions, standing hip flexions, and standing calf raises using ankle cuff weights. (Isotonic exercise)	Started with 5–10 lb ankle weights, progressing from 2 sets of 8 reps to 3 sets of 12 reps over 6 weeks, with weight increases in the final 4 weeks.	2 times/week	10 weeks	Yes
